# Distinct transcriptional and epigenomic programs define Hofbauer cells in term placenta

**DOI:** 10.1172/jci.insight.195801

**Published:** 2025-12-23

**Authors:** Benjámin R. Baráth, Dóra Bojcsuk, Krisztian Bene, Noemí Caballero-Sánchez, Tímea Cseh, João CR. de Freitas, Petros Tzerpos, Marta Toth, Zhonghua Tang, Seth Guller, Zoárd Tibor Krasznai, Patrícia Neuperger, Gabor J. Szebeni, Gergely Nagy, Tamás Deli, Laszlo Nagy

**Affiliations:** 1 Department of Biochemistry and Molecular Biology, Faculty of Medicine, and; 2 Doctoral School of Molecular Cell and Immune Biology, University of Debrecen, Debrecen, Hungary.; 3 Institute for Fundamental Biomedical Research, Johns Hopkins All Children’s Hospital, St. Petersburg, Florida, USA.; 4 Department of Immunology, Faculty of Medicine, University of Debrecen, Debrecen, Hungary.; 5 Department of Obstetrics, Gynecology and Reproductive Sciences, Yale School of Medicine, New Haven, Connecticut, USA.; 6 Department of Obstetrics and Gynecology, Faculty of Medicine, University of Debrecen, Debrecen, Hungary.; 7 Laboratory of Functional Genomics, Core Facility, HUN-REN Biological Research Centre, Szeged, Hungary.; 8 Department of Internal Medicine, Hematology Centre, Faculty of Medicine, University of Szeged, Szeged, Hungary.; 9 Departments of Medicine; Pediatrics; Physiology, Pharmacology and Therapeutics; and Biomedical Engineering, Johns Hopkins University School of Medicine, Baltimore, Maryland, USA.

**Keywords:** Immunology, Reproductive biology, Macrophages

## Abstract

Hofbauer cells (HBCs) are fetal macrophages located in the placenta that contribute to antimicrobial defense, angiogenesis, tissue remodeling, and metabolic processes within the chorionic villi. Although their roles in placental biology are increasingly recognized, the mechanisms that regulate HBC identity and function are not yet fully defined. This study aimed to define the core transcriptomic and epigenomic features of HBCs in term placentas and to examine their capacity for transcriptional responsiveness and phenotypic variation. Using chromatin accessibility profiling and bulk RNA-seq, we found that HBCs exhibit a unique gene expression and chromatin accessibility profile compared with other fetal and adult macrophages. We identified a coordinated transcriptional network involving nuclear receptors (NRs) NR4A1–3, the glucocorticoid receptor, and RFX family members (RFX1, RFX2, RFX5) that appears to shape HBC identity, particularly through pathways linked to lipid metabolism and angiogenesis. Although exploratory in nature, in vitro stimulation studies showed that HBCs exhibited increased transcriptional activity in response to combined IL-4 and rosiglitazone treatment, including induction of the lipid transporter CD36. Mass cytometry analysis revealed surface markers indicative of both immature and mature macrophage states. These results together indicate that HBCs are a distinct and diverse population of macrophages with a specialized, adaptable regulatory program in the human placenta.

## Abstract 2

Our study defines unique regulatory programs of placental Hofbauer cells, advancing understanding of their role in pregnancy health and potential disease.

## Graphical abstract

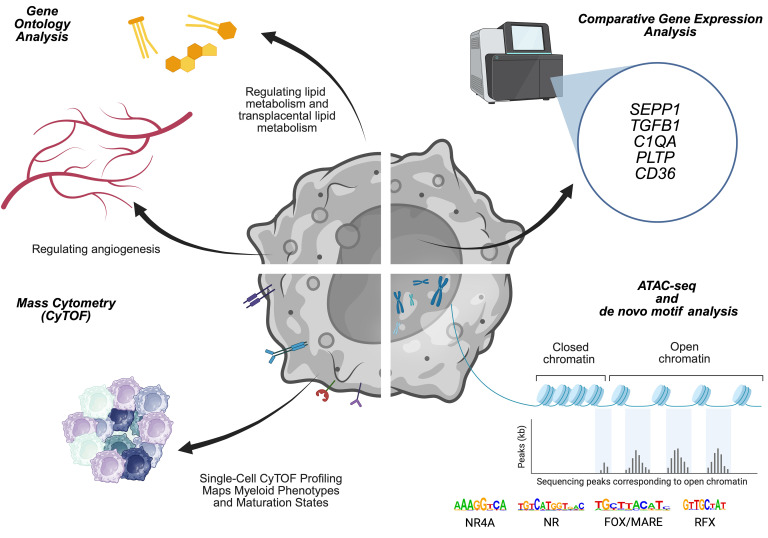

## Introduction

Macrophages are a heterogeneous population of immune cells of myeloid origin that constantly fine-tune their functional state and that of surrounding cells in response to changes in the tissue environment (1). Macrophages originate from erythroid-myeloid progenitors or hematopoietic stem cells, with tissue-specific resident macrophages primarily derived from yolk sac–derived erythroid-myeloid progenitors during development (2) and hematopoietic stem cell–derived macrophages recruited later in life associated with aging and diseased or chronic inflammatory states. Lineage-determining transcription factors, along with other signals from the surrounding cell niche, drive cell type–specific tissue-resident macrophage differentiation during organogenesis. However, the specific signals and mechanisms inducing and maintaining tissue macrophage identity and function have yet to be determined for each macrophage subtype.

The human placenta is a transient yet essential and highly versatile organ, playing a vital role in the progression of pregnancy. It forms a vital interface between the mother and fetus, facilitating the transport of oxygen, nutrients, and waste products, while also producing hormones crucial for pregnancy and fetal development. It performs these functions through its highly specialized microarchitecture and a distinct, placenta-specific set of cells. Fetal placental macrophages, also known as Hofbauer cells (HBCs), are large (10–30 μm in diameter), vacuolated, and granulated cells found in the chorionic villi of the placenta (3, 4) in proximity to villous endothelial cells, fibroblasts, and the different subtypes of trophoblasts. Functional and gene expression studies have shown that HBCs exhibit a high level of phagocytosis and play an essential role in protecting against pathogens and in placental morphogenesis (e.g., vasculogenesis, angiogenesis), maintaining placental tissue homeostasis (5–8). The latest findings suggest that HBCs are derived from placental erythromyeloid precursors throughout pregnancy, with no apparent monocyte-derived supplementation under homeostatic conditions (9). We refer to previously written articles focusing on HBCs in various pregnancy complications (7, 8, 10, 11).

The limited epigenetic studies available on HBCs shed light on their origin and the mechanism underlying the differences in HLA-DR expression of HBCs during pregnancy. Thomas and colleagues reported that although first-trimester HBCs lack HLA-DR expression, third-trimester HBCs exhibit variable HLA-DR levels. Additionally, HBC DNA methylation profiles remain consistent throughout pregnancy, suggesting negligible monocyte contribution (9). Methodological advances in recent years have made it possible to study cellular heterogeneity at the fetal-maternal interface (12–14). Given the structural and physiological differences between humans and other species, the relevance of animal models, especially mouse models, is often debated in the context of reproductive research focusing on the human placenta. One intriguing study found a transcriptomic correlation between mouse placental macrophages, human HBCs, and microglia cells, suggesting that HBCs could serve as a biomarker for brain damage susceptibility (15).

Complications such as preeclampsia, villitis of unknown etiology, fetal growth restriction, and preterm labor are frequently associated with abnormalities in placental vascular development, inflammation, and disruptions in maternal-fetal immune regulation, processes in which HBCs are thought to contribute. However, the regulatory networks that define HBC identity, function, and phenotype under physiological conditions remain incompletely understood. To address this gap, we performed a systematic molecular characterization of primary HBCs isolated from uncomplicated term placentas. Using an integrative multiomics approach, including bulk RNA-seq, assay for transposase-accessible chromatin sequencing (ATAC-seq), cytometry by time-of-flight (CyTOF), and in vitro stimulation experiments, we sought to define the transcriptional and chromatin accessibility landscape of HBCs and assess their potential for regulatory responsiveness and phenotypic variation. This approach provides a foundational step toward characterizing the transcriptional and regulatory landscape of purified HBCs in late gestation and may serve as a useful reference for future investigations into placental immune function and related pathologies.

## Results

### Purification and phenotypic characterization of term HBCs.

**Figure 1:**
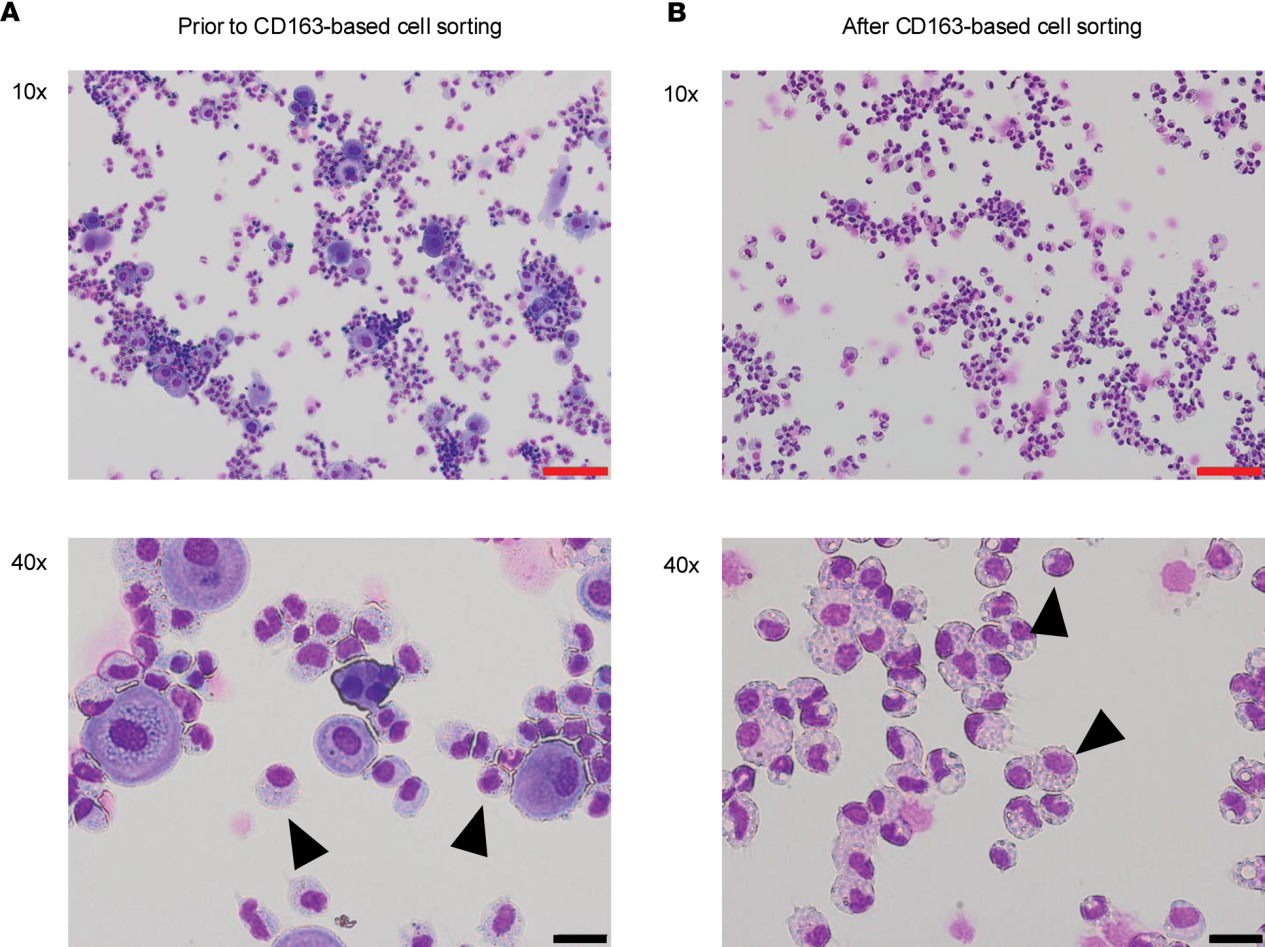
Enrichment of HBCs by CD163-based cell sorting. Cytospin microscopic pictures showing HBC isolates (**A**) before and (**B**) after CD163-based sorting. Arrowheads point at HBCs. Red scale bars: 100 μm. Black scale bars: 20 μm.

Five eligible term placentas were collected, each within 20 minutes of elective cesarean section performed without labor at the University of Debrecen, Department of Obstetrics and Gynecology (Table 1). HBCs were isolated using negative selection for CD10 and EGFR, and then sorted based on CD163 expression as described previously (Table 2) (7). Prior to CD163 sorting, 70%–80% of the cells showed typical pleomorphic macrophage morphology with characteristic granular, vacuolar, and foamy cytoplasm. In the remaining 20%–30%, we detected large contaminating cells (data not shown; Figure 1A). The contamination was reduced upon sorting down to approximately 10% (Figure 1B and Supplemental Figure 1B; supplemental material available online with this article; https://doi.org/10.1172/jci.insight.195801DS1). Our gating strategy used for FACS is depicted in Supplemental Figure 1. Our single-cell RNA-seq supported the macrophage identity of our isolates (Supplemental Figure 2). Considering the possibility of contamination with maternal decidua macrophages, we benefited from the presence of male newborns (*n* = 3), which allowed us to estimate the percentage of impurity by tracking the extent of female material through sex-specific genes. Analyzing X-linked gene expression (i.e., X inactive specific transcript [*XIST*] expression), we estimated the potential contamination to be between 2% and 9% (Supplemental Table 1). The purity of the isolation was further supported by the principal component analysis plot comparing our isolates with maternal decidual and placental macrophages of first and third trimesters (Supplemental Figure 3) (13, 16). The high-yield isolation of HBCs allowed us to ask questions regarding the molecular characteristics, such as global gene expression, of this distinct cell type.

### HBCs have a distinct gene expression landscape compared with other fetal and adult macrophages.

Next, we focused on the gene expression (bulk RNA-seq) profile of isolated HBCs and compared them with 15 fetal and adult monocyte and macrophage subpopulations: adult Kupffer cells (KCs) (17), monocyte-derived macrophages (MDMs) (18), alveolar macrophages (AMs) (19), adult microglia (aMG) (20), fetal microglia (fMG) (21), fetal liver macrophages (fLMs) (21), mononuclear and multinucleated osteoclasts (22), osteoclast precursors (23), spleen macrophages (24), intestinal macrophages (25), and skin macrophages (26), as well as spleen monocytes (24), blood monocytes (24), and cord blood monocytes (CBMos) (27).

**Figure 2:**
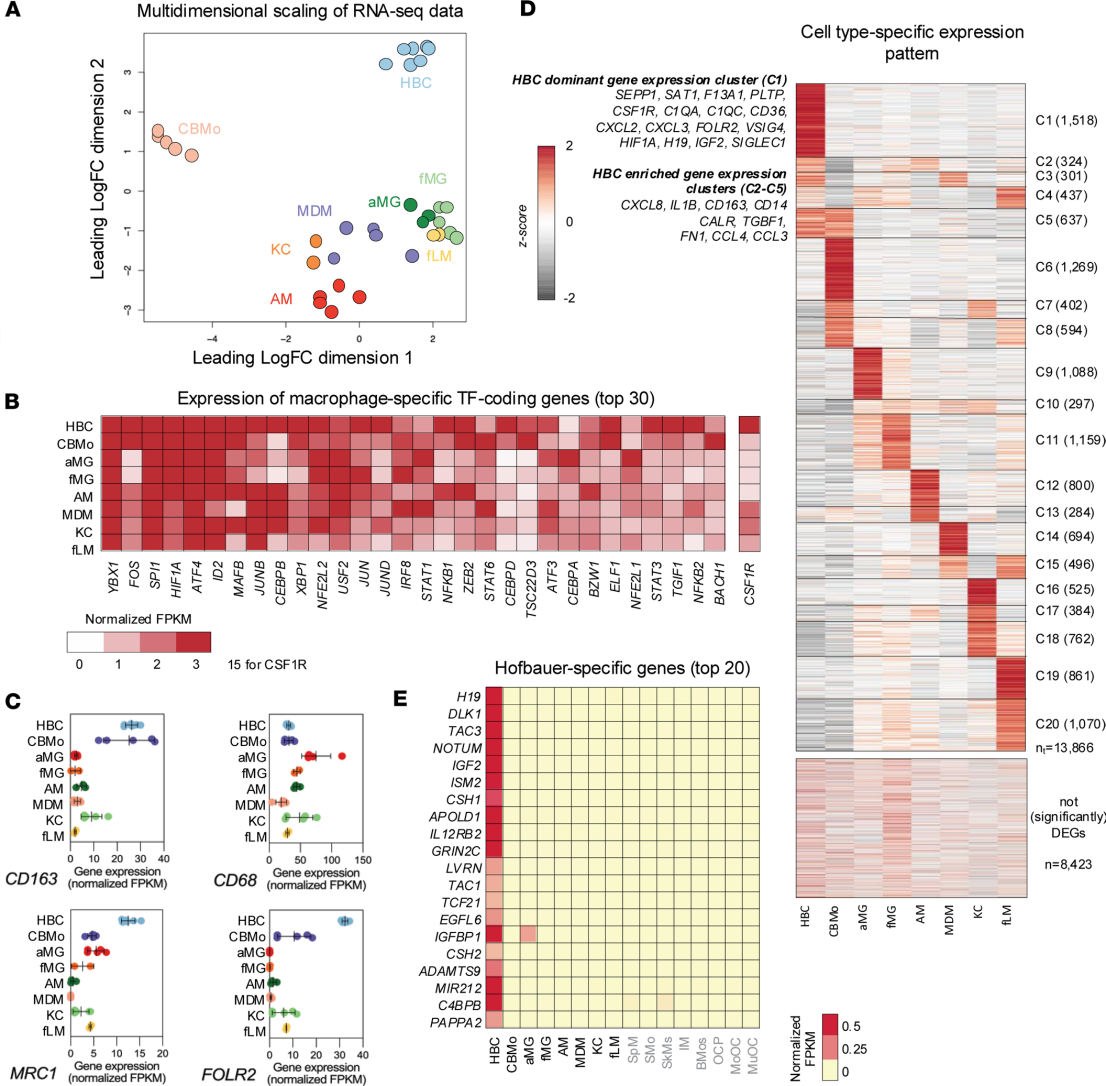
Comparative transcriptomic profiling of HBCs. (**A**) The multidimensional scaling plot represents the Euclidean distances between the gene expression patterns of the 8 cell types indicated and also between their replicates. (**B**) The heatmap shows the average normalized gene expression of the top 30 macrophage-specific transcription factor–coding genes and the CSF1R gene in the 8 cell types. (**C**) Scatterplots showing normalized gene expression values (FPKM) for HBC marker genes, including *CD163*, *CD68*, *MRC1*, and *FOLR2* across 8 cell types. Points represent individual replicates; horizontal bars indicate the mean, and error bars represent SD. (**D**) The row-normalized, clustered heatmap represents the cell subtype–specific (*P* ≤ 0.05; fold difference ≥ 2) average of the normalized gene expression patterns and the non-DEGs between them. DEGs, differentially expressed genes. (**E**) The heatmap represents the average normalized expression of the top 20 HBC-specific marker genes. The corresponding FPKM scale is shown at the lower left corner of the heatmap. KC, Kupffer cell; MDM, monocyte-derived macrophage; AM, alveolar macrophage; aMG, adult microglia; fMG, fetal microglia; fLM, fetal liver macrophage; MoOC, mononuclear osteoclast; MuOC, multinucleated osteoclast; OCP, osteoclast precursor; SpM, spleen macrophage; IM, intestinal macrophage; SkM, skin macrophage; SMo, spleen monocyte; BMo, blood monocyte; CBMo, cord blood monocyte.

Multidimensional scaling of RNA-seq data revealed that all samples of HBC populations clustered together rather tightly, and they appeared to be distinct from other healthy fetal and adult macrophage populations (Supplemental Figure 4A). For further, more in-depth analysis, we only kept those cell types that had a fetal counterpart (CBMo, aMG, fMG, KC, and fLM, as well as those whose transcriptional regulation we later examined together with that of the HBCs (AM and MDM; Figure 2A). These comparisons showed that the HBC populations from our donors were similar to each other, with little patient-to-patient variability as far as their global gene expression was concerned, and that HBCs’ gene expression profile was distinct, as they were clustered far from other fetal and adult macrophage populations. Moreover, HBCs displayed a distinct gene expression profile, clustering far from other fetal and adult macrophage populations. The low intersample variability is also illustrated by the correlation plot with Pearson’s correlation values in Supplemental Figure 4B and the sample-to-sample distance heatmap in Supplemental Figure 4C. This raises, at least formally, the question of whether these cells meet the criteria for being bona fide macrophages.

To support the claim that the isolated cells were indeed macrophages, we used a list of macrophage-specific transcription factors and plotted the average expression of the top 30 genes encoding these factors (Figure 2B) (28). HBCs showed high levels of expression genes encoding these factors (i.e., transcription factor PU.1 [*PU.1*], Y-box binding protein 1 [*YBX1*], certain basic helix-loop-helix (bHLH) proteins such as hypoxia-inducible factor 1 subunit alpha [*HIF1A*], inhibitor of DNA binding 2 [*ID2*], and upstream transcription factor 2 [*USF2*], as well as activating transcription factor 3 [*ATF3*] and 4 [*ATF4*] and IFN regulatory factor 8 [*IRF8*]); in addition, the essential macrophage marker colony-stimulating factor 1 receptor [CSF1R] was expressed at a high level in HBCs, further establishing that the isolated cells were indeed macrophages. This comparison also showed key differences in the relative expression of these genes when HBCs were compared with other fetal and adult macrophage subpopulations. For example, HBCs expressed CCAAT/enhancer-binding protein delta (*CEBPD*) as opposed to *CEBPA*, which is highly enriched in microglia, and *CEBPB*, which is expressed primarily in AMs, KCs, and MDMs. Figure 2C highlights the high expression of genes that are considered as HBC marker genes within the context of the fetal-maternal interface (29).

### Distinct gene expression pathways are enriched in HBCs.

**Figure 3:**
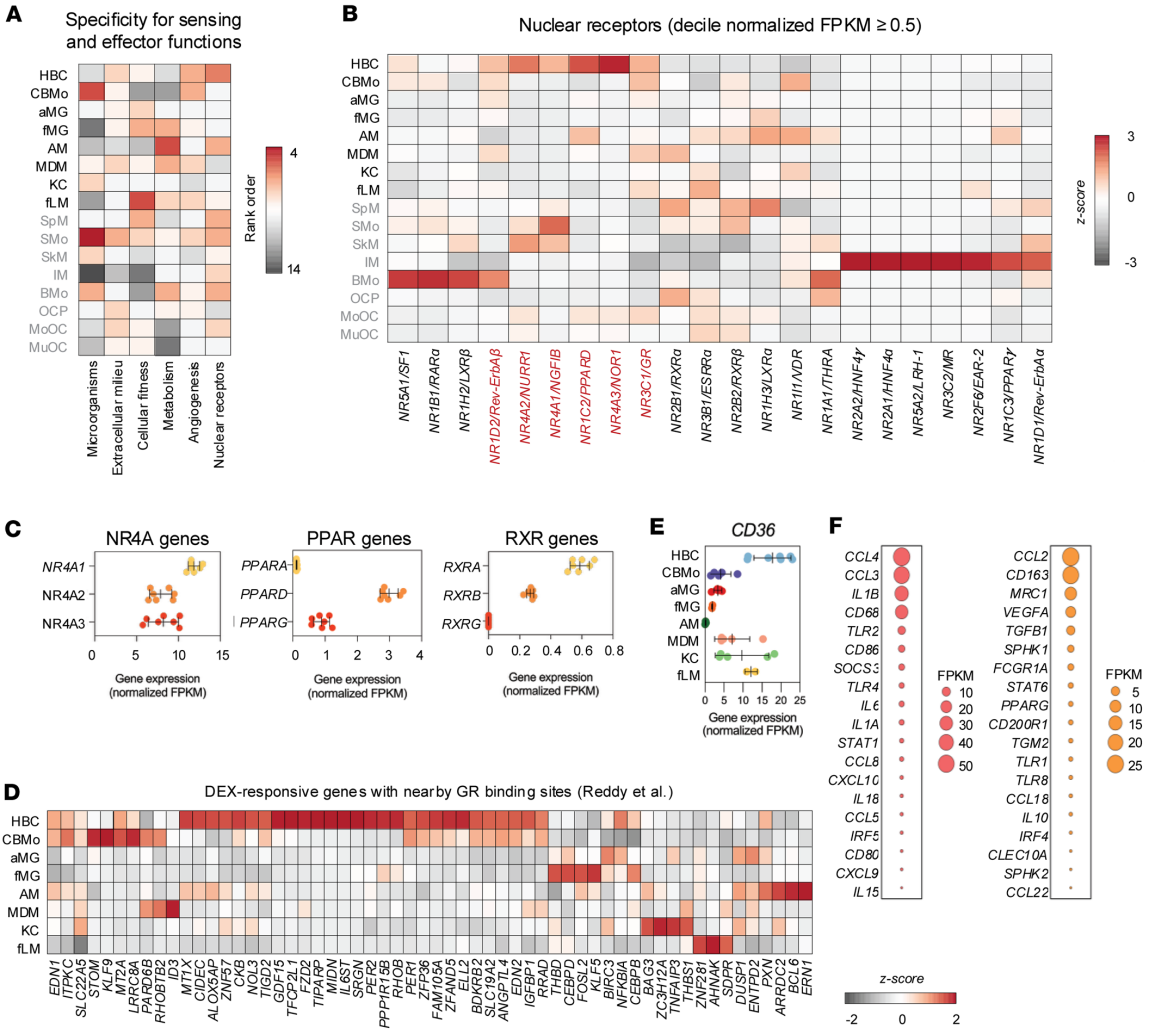
Transcriptional profiling of nuclear receptor expression and functional specificity in the included macrophage populations. (**A**) The heatmap represents the significance of the genes (in rank order) of the 6 highlighted sensing and effector functions in the 16 cell types. Genes with an expression higher than 0.5 FPKM (after normalization) in any cell type were considered. (**B**) The row-normalized heatmap depicts the gene expression of the nuclear receptor superfamily members in the 16 cell types. (**C**) Scatterplots showing normalized gene expression values (FPKM) for NR4A, PPAR, and RXR family members in sequenced HBC samples. Points represent individual replicates, with horizontal bars indicating the mean and error bars representing SD. (**D**) The row-normalized heatmap depicts the expression of DEX-responsive genes with nearby GR binding sites, which were previously described (32). (**E**) Scatterplot showing normalized gene expression values (FPKM) of *CD36*. Points represent individual replicates, with horizontal bars indicating the mean and error bars representing SD. (**F**) Bubble plot shows the expression levels (FPKM) of M1 macrophage marker genes (red, left panel) and M2 macrophage marker genes (orange, right panel) in HBCs. The size of each bubble corresponds to the expression value (FPKM) as indicated in the scale legends. Larger bubbles represent higher transcript abundance. KC, Kupffer cell; MDM, monocyte-derived macrophage; AM, alveolar macrophage; aMG, adult microglia; fMG, fetal microglia; fLM, fetal liver macrophage; MoOC, mononuclear osteoclast; MuOC, multinucleated osteoclast; OCP, osteoclast precursor; SpM, spleen macrophage; IM, intestinal macrophage; SkM, skin macrophage; SMo, spleen monocyte; BMo, blood monocyte; CBMo, cord blood monocyte.

Since gene expression analysis verified the purity and identity of the isolated cells as fetal macrophages, we considered the different biological processes regulated by transcription in HBCs. We systematically compared the bulk RNA-seq profiles obtained from HBCs, CBMos, aMG, fMG, AMs, MDMs, KCs, and fLMs and identified 20 gene clusters representing 13,866 differentially expressed genes (DEGs; Figure 2D). Additionally, we found 8,423 genes expressed at similar levels (no significant statistical difference between the groups) in all these cell types (Figure 2D). Clusters C1–C5 contained genes enriched in HBCs, with cluster 1 (C1) being primarily HBC dominant, while clusters 2–5 (C2, C3, C4, C5) also showed considerable expression in other cell types. CD36, HIF1A, selenoprotein P (*SEPP1*), *CSF1R*, complement C1q A chain and C chain (*C1QA* and *C1QC*, respectively), V-set and Ig domain containing 4 (*VSIG4*), phospholipid transfer protein (*PLTP*), and the HBC marker folate receptor beta (*FOLR2*) are all genes that were highly expressed in HBCs and found in C1. Highly expressed genes in C2–C5 included *CCL4*, *CCL3*, *CXCL8*, *CD163*, *CD14*, *IL1B*, and fibronectin 1 (*FN1*). Among the genes showing absolute HBC specificity were H19 imprinted maternally expressed transcript (*H19*), delta-like noncanonical Notch ligand 1 (*DLK1*), tachykinin precursor 1 and 3 (*TAC1* and *TAC3*), *IGF2*, isthmin 2 (*ISM2*), chorionic somatomammotropin hormone 1 and 2 (*CSH1* and *CSH2*), apolipoprotein L domain containing 1 (*APOLD1*), IGF binding protein 1 (*IGFBP1*), and pappalysin 2 (*PAPPA2*; Figure 2E). In addition, the 1,518 genes of C1 were enriched in pathways related to tissue and embryo morphogenesis, heart and skeletal muscle development, epithelial cell proliferation, and *VEGFA*-*VEGFR2* signaling controlling angiogenesis (Supplemental Figure 4D). In order to compare tissue macrophage functions in a more systematic and potentially functional manner, we took curated gene sets representing key macrophage sensing and effector functions, such as cellular fitness, extracellular milieu, metabolism, nuclear receptors (NRs), the detection of and response to microorganisms as outlined by Lazarov et al. (30) (Figure 3A), and compared the expression of these key gene sets along with genes responsible for angiogenesis (derived from the PANTHER Pathways dataset). As shown in Figure 3A, HBCs were distinct in having higher expression levels of angiogenetic, extracellular milieu, cellular fitness, and NR signaling genes. Example genes for these functions are depicted in Figure 3B and Supplemental Figures 5 and 6. There was a lower expression of genes associated with the response to microbes and metabolism. Despite the low ranking of metabolism, genes playing a role in lipid metabolism and transport were highly represented in C1 and C2 (e.g., *CD36*, *PLTP*, and acyl-CoA synthetase long chain family member 1 [*ACSL1*]). NR signaling and lipid metabolism regulation were functionally linked: *NR4A* genes, *PPAR*s, retinoid X receptor alpha (*RXRA*), and glucocorticoid receptor (*GR*) were enriched in HBCs (Figure 3, B and C). Given the prior in vitro demonstration of HBC dexamethasone (DEX) responsiveness by Tang et al. (31), we investigated GR-associated gene sets in this cell type, finding that the majority of DEX-responsive genes with nearby GR binding sites showed higher expression in HBCs compared with the other examined cell types (Figure 3D) (32). Importantly, the expression of the NR PPAR-regulated scavenger and the fatty acid receptor *CD36* was particularly high in HBCs, suggesting a key role for this latter molecule (Figure 2D and Figure 3E). Finally, given the low ranking of the antimicrobial function in Figure 3A, we assessed whether HBCs represent a cell type already committed to one of the prototypic macrophage polarization paradigms (M1 or M2). Examining a set of selected polarization markers (33–36), we found that HBCs showed a mixed polarization phenotype with high expression levels of genes of certain M1 (*CCL4*; *CCL3*, *IL-1B*) and M2 markers (*CD163*, mannose receptor C-type 1 [*MRC1*], *CD36*); concurrently, prototypic marker genes had low expression (*CD80* and transglutaminase 2 [*TGM2*]) (Figure 3F). Collectively, these findings suggest that HBCs actively shape, sense, and interact with their surroundings; are particularly responsive to lipid-soluble molecules; express high levels of CD36; and likely play a major role in regulating angiogenesis. Next, we were interested to find out how chromatin-level regulation supports this transcriptional and likely functional diversity in HBCs.

### HBCs have a distinct chromatin openness profile and have a cell type–specific set of promoters and enhancers.

Next, we decided to carry out ATAC-seq experiments to determine the chromatin accessibility in HBC isolates and compare those with published data on human AMs from the lung (19) and MDMs (18), representing a cell type typically responding to inflammatory stimuli. The correlation heatmap shows uniformly high sample-sample similarity among HBC samples and strong correlations across patients, indicating high data consistency and reproducibility (Supplemental Figure 7A).

**Figure 4:**
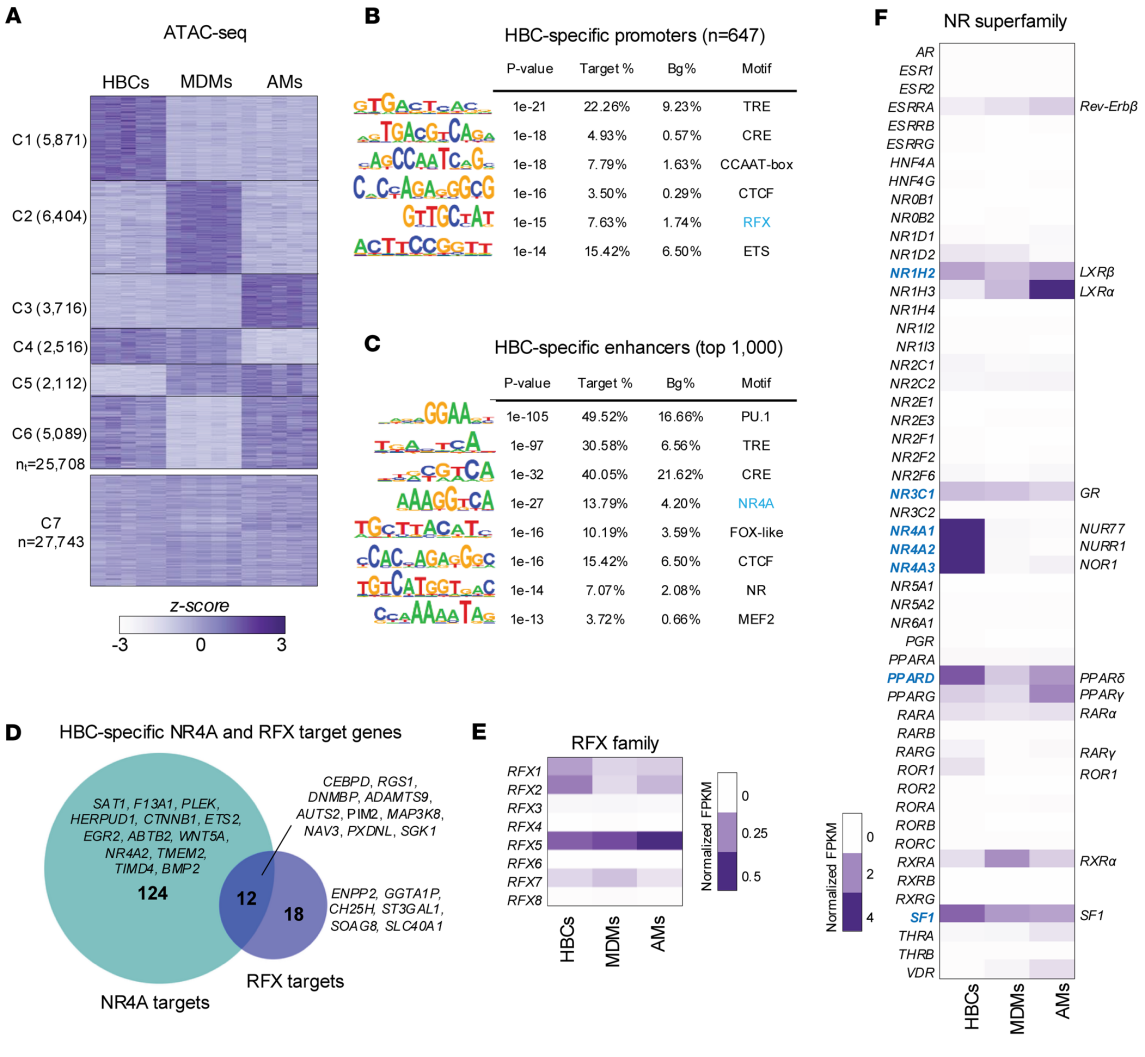
Chromatin accessibility and enrichment of transcription factor motifs delineate HBC-specific regulatory programs. (**A**) The row-normalized, clustered heatmap represents the coverage values of those differentially accessible chromatin regions (*P* ≤ 0.05; fold difference ≥ 2) specific for the HBCs, MDMs, or AMs, or their combinations (C1–C6). It also depicts the non-differentially accessible chromatin regions (C7). (**B**) The de novo motif hits of the HBC-specific TSS-proximal (promoter) (*n* = 647) and (**C**) the top 1,000 TSS-distal (enhancer) regions. Logos of the significantly enriched motifs are presented along with the *P* value rank; Bg%, background percentage. (**D**) The proportional Venn diagram represents the overlap between the NR4A and RFX target genes that display significantly higher expression and where NR4A and RFX motifs are located within more accessible chromatin regions in HBCs. The heatmaps represent (**E**) the average normalized gene expression of the members of the *RFX* and (**F**) the nuclear receptor (super)families in the HBCs, MDMs, or AMs. The row-normalized heatmap represents the coverage values.

As shown in Figure 4A (C7), 27,743 genomic regions demonstrated similar chromatin accessibility between HBCs, MDMs, and AMs. However, we were able to detect 25,708 genomic regions that showed significant differences (*P* ≤ 0.05; FC ≥ 1.5) in chromatin accessibility (Figure 4A). These regions could be grouped into 6 clusters: 3 of them were cell type specific (C1–C3), and the other 3 were characteristic of 2 cell types each (C4–C6). To uncover the DNA-binding motifs at these distinctly open genomic regions, we carried out de novo motif enrichment analyses separately for the TSS-proximal (promoter) and TSS-distal (enhancer) genomic regions (Figure 4, B and C).

TSS-proximal regions of C1 (*n* = 647), which were distinct to HBCs, were enriched in certain motifs, such as the motifs of the TPA-response element binding motif (TRE; 22.26%), cAMP response element binding motif (CRE; 4.93%), E26 transformation-specific (ETS; 15.42%) family motif, and CCAAT-boxes (7.79%) (Figure 4B). However, GC-boxes, which were enriched in AM- and MDM-specific TSS-proximal regions (C2 and C3), were generally absent in HBCs. Interestingly, the regulatory factor X binding motif (RFX) was present at 7.63% of peaks in HBCs and enriched in the common regions between HBCs and AMs (C4, 9.12%) (Figure 4B and Supplemental Figure 7B).

The significantly more open TSS-distal regions of all 3 macrophage subtypes were enriched for myeloid-specific motifs, such as PU.1, TRE, CRE, or runt-related transcription factor (RUNX) binding motif. Early growth response (EGR; 24.03%) motifs and the motifs of certain bHLH proteins, such as activating enhancer-binding protein 4 (AP-4; 20.34%), appeared to be specific for MDMs, whereas AM could be characterized by the presence of the already-described CEBP (33.81%). HBCs have shown distinct enrichments for NRs such as NR4A (e.g., NUR77 [13.79%]; Figure 4C and Supplemental Figure 7C) and a sequence motif containing a potential MAF binding site (MARE half site; 10.19%), which only differs from the FOX motif by a single nucleotide (37–39).

**Figure 5:**
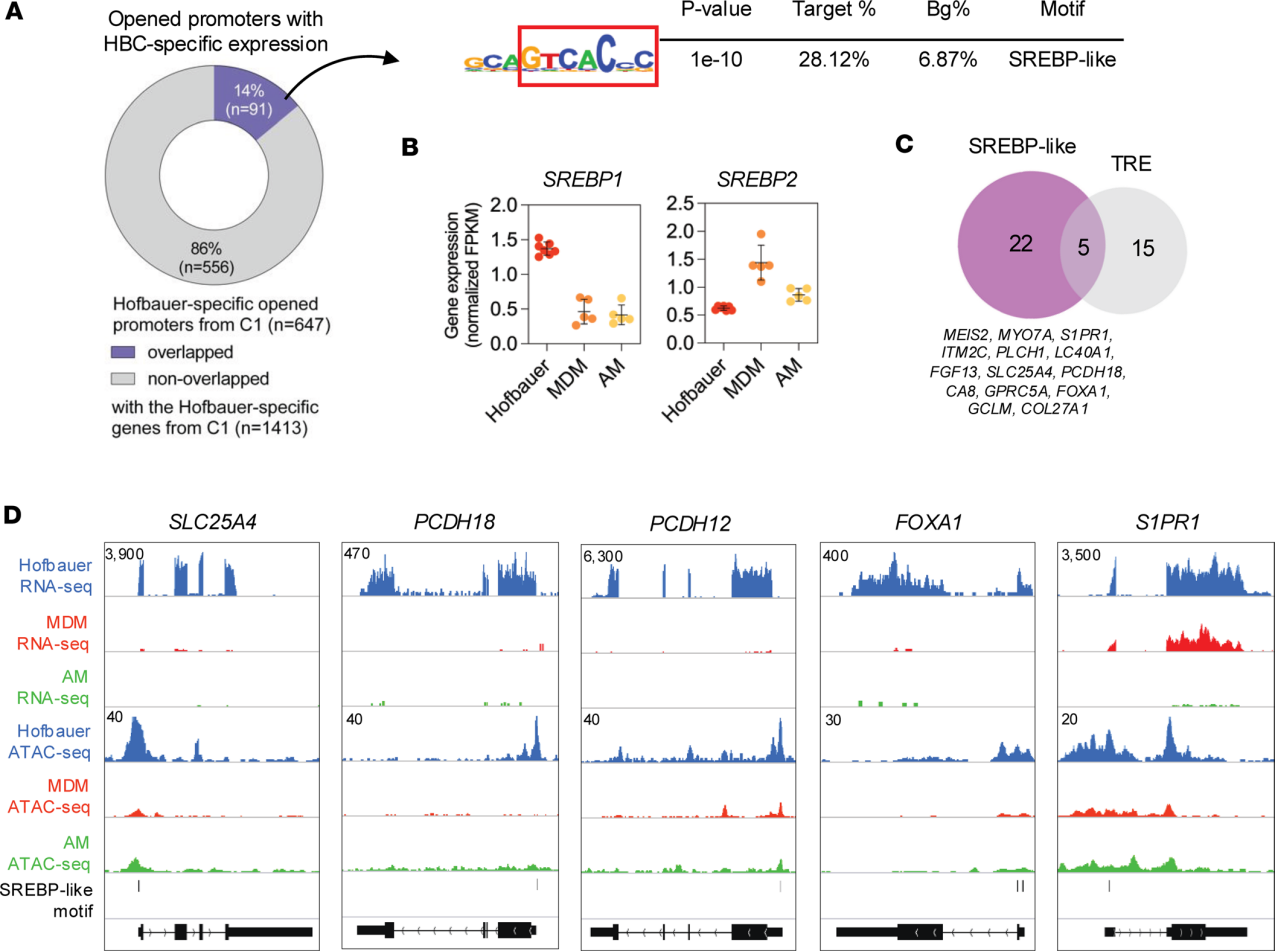
SREBP-like motif enrichment and target gene activity in HBCs. (**A**) SREBP-like motif enriched in promoters of genes that are upregulated and have more increased promoter accessibility in HBCs. (**B**) Gene expression of the SREBP1 and SREBP2 genes in HBCs, MDMs, and AMs. (**C**) Number of mapped SREBP-like and TRE motifs in open promoters of 91 HBC-specific genes. Selected genes with SREBP-like motifs are highlighted. (**D**) The genome browser view of RNA-seq and ATAC-seq coverages of HBC, MDM, and AM cells on the SLC25A4, PCDH18, PCDH12, FOXA1, and S1PR1 loci. The coverage files of the replicates are overlayed, and the interval scales are highlighted in all cases.

Combining the bulk RNA-seq and ATAC-seq data allowed us to associate the enriched motifs with their potential regulator transcription factor(s) using gene expression data. Figure 4D depicts NR4A and RFX target genes that display significantly higher expression (RNA-seq C1) and where NR4A and RFX motifs are located within more accessible chromatin regions in HBCs (ATAC-seq C1). Integrative Genomics Viewer genome browser views of representative NR4A and RFX target genes are presented in Supplemental Figure 8. As shown in Figure 4E, several members of the RFX transcription factor family are expressed in HBCs, such as *RFX1*, *RFX2*, and *RFX5*, of which the latter has the highest expression level. As Figure 3B depicts and Figure 4F further outlines, numerous NRs show high expression in HBCs, out of which NR4A family members are definitely worth highlighting, given that their binding motif also shows enrichment (Figure 4C). Both Figure 3B and Figure 4F underscore the high expression of *GR*. Furthermore, by using a dataset representing GR target genes, we recognized that the GR is likely to be active due to the high induction levels of many glucocorticoid target genes (Figure 3D) (32). These data, combined with the gene sets presented in Figure 3, A and B, and Figure 4F, strongly suggest that HBCs have active metabolic signaling involving NRs and lipids. Interestingly, by overlapping the HBC-specific opened TSS-proximal (promoter) regions and the HBC-specific genes from C1 of Figure 2D, only a small subset of opened promoters could be associated with HBC-specific gene expression (Figure 5A). We identified 91 promoters, resulting in a 28.12% enrichment of the SREBP-like motif. Based on gene expression data, SREBP1 will most likely bind this motif in HBCs (Figure 5B). Since the enriched motif contains the sequence “TCA,” to rule out the possibility that an incomplete or distorted TRE motif was enriched, we mapped the TRE motif and found no significant presence in the 91 promoter regions (Figure 5C). Promoters with an SREBP-like sequence are found in genes such as *SLC25A4*, as well as *PCHD18*, *PCDH12*, *FOXA1*, and *S1PR1* (Figure 5D). These results further underscore the role of HBCs in lipid and fatty acid metabolism; however, this is likely a subset and not a complete set of gene-regulatory region pairs due to the statistical approach.

### HBCs exhibit synergistic transcriptional activation upon exposure to IL-4 and rosiglitazone.

**Figure 6:**
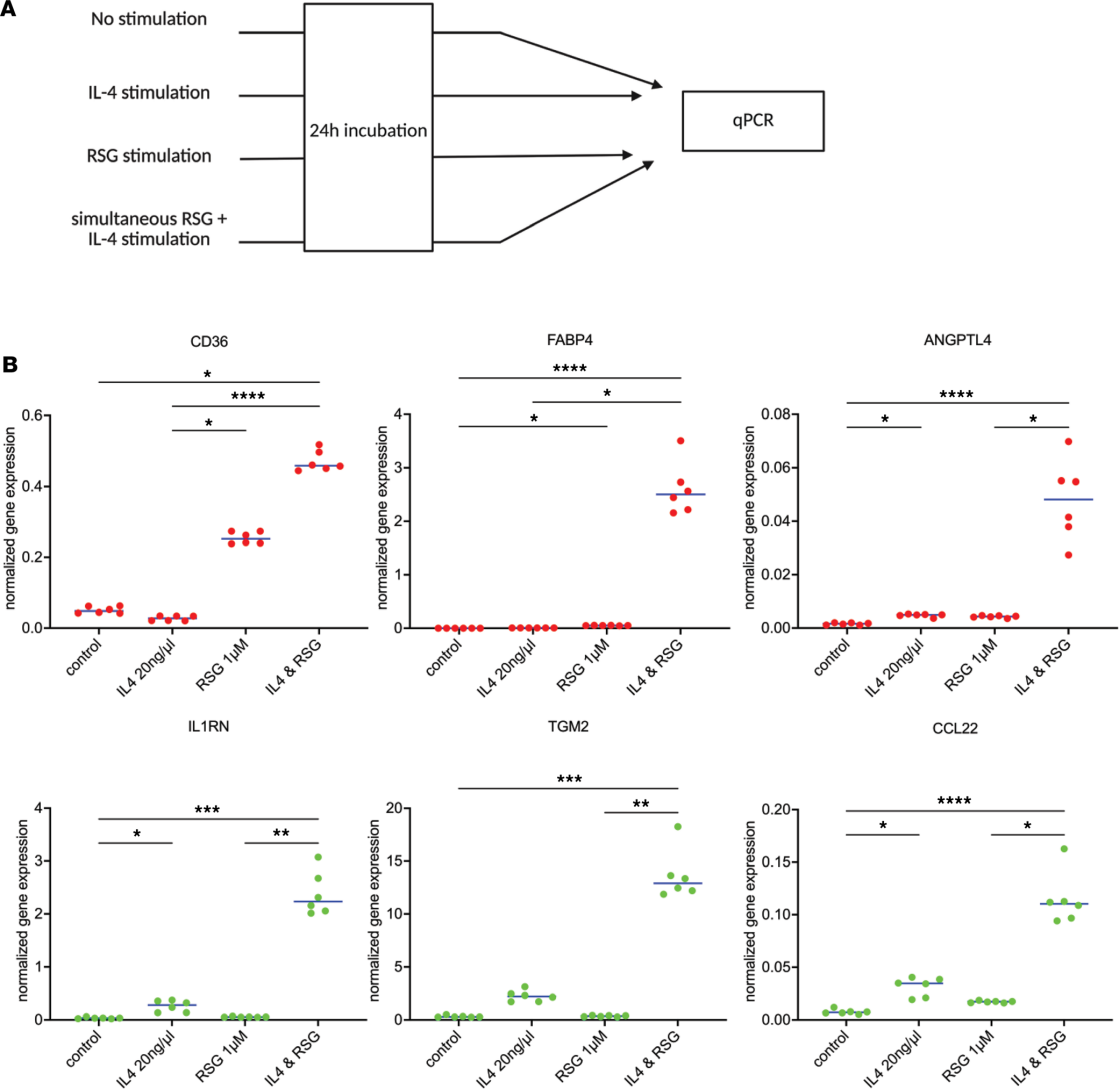
Synergistic effects of rosiglitazone and IL-4 on gene expression in in vitro stimulation assays. (**A**) Experimental outline of the in vitro induction studies. (**B**) Normalized mRNA expression levels after in vitro rosiglitazone and IL-4 induction, measured using PCR. Canonical PPARγ target genes are in red, and IL-4–induced genes are in green. Blue lines represent the medians of individual values. Pairwise comparisons were made using Kruskal-Wallis tests followed by Dunn’s post hoc tests. The figure depicts replicates from the cells of the same placenta (placenta number 5); 1 dot represents 1 parallel measurement. Canonical PPARγ target genes are indicated in red, canonical IL-4–induced genes are indicated in green. Statistical significance is indicated as follows: *P < 0.05, **P < 0.01, ***P < 0.001, ****P < 0.0001. Supplemental Figure 9 depicts the PCR data of 4 placentas.

These findings prompted us to examine the in vitro programmability of HBCs using an established signaling paradigm as a proof of concept. Given that CD36 is a key lipid mediator and scavenger receptor with likely functions regarding lipid handling, we decided to test whether its expression can be modulated using the well-established model of IL-4–induced and ligand-activated PPARγ (Figure 6) (40). Therefore, we set up in vitro cultures and treated cells with IL-4 and/or rosiglitazone (Figure 6A). We present a representative experiment of biological replicates from HBCs isolated from a single placenta (Figure 6B). Treating cells with these ligands resulted in synergistic gene expression of *CD36*, angiopoietin-like 4 (*ANGPTL4*), and IL-1 receptor antagonist (*IL1RN*), providing proof-of-concept data for the programmability of HBCs. Similar results were obtained from other placentas (Supplemental Figure 9).

### Single-cell CyTOF profiling maps myeloid phenotypes and maturation states in HBCs.

Finally, we performed CyTOF to complement the gene expression data with qualitative and quantitative single-cell protein expression data and to examine the potential degree of single-cell heterogeneity in our HBC isolates.

**Figure 7:**
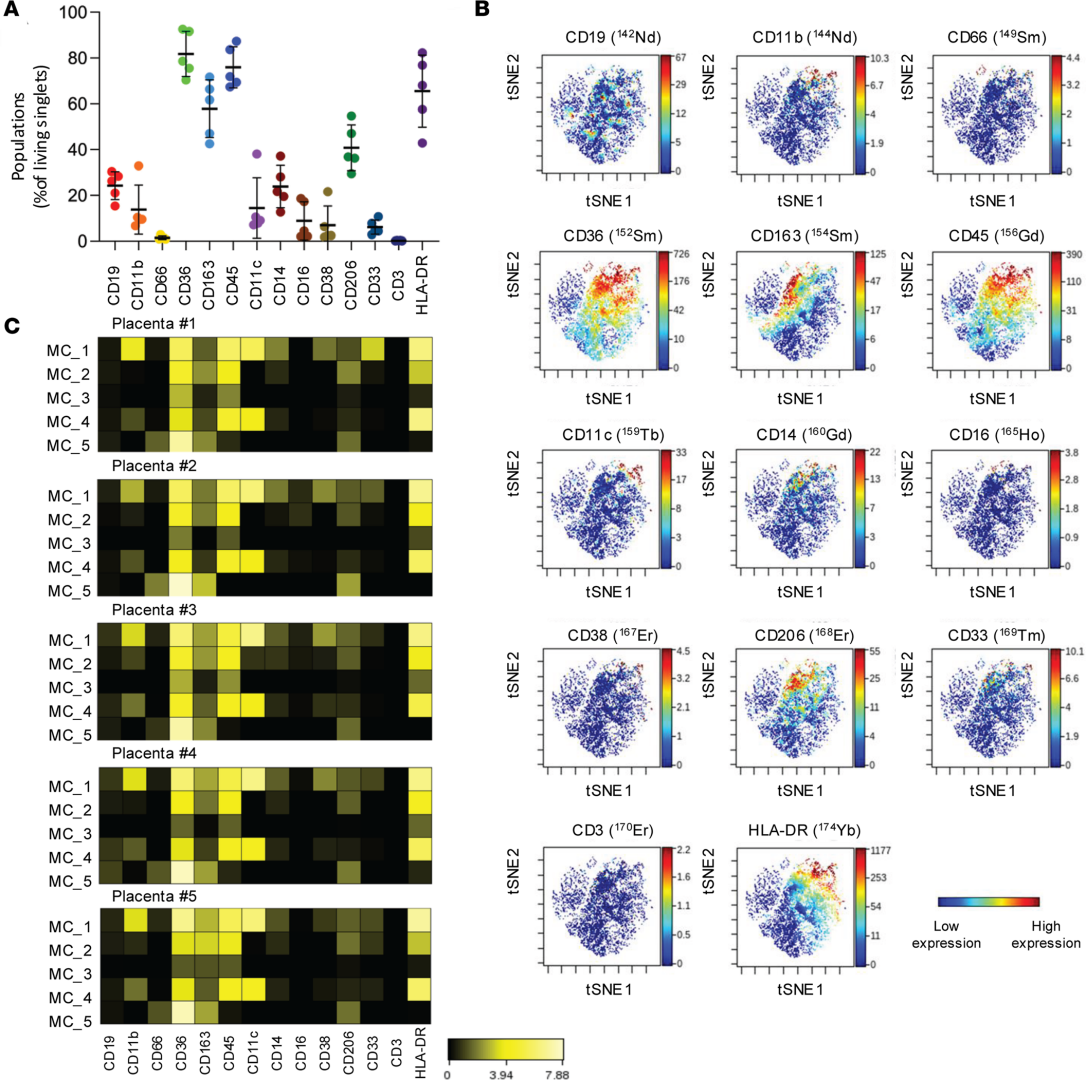
Single-cell heterogeneity of HBCs revealed by multidimensional immunophenotyping using CyTOF. (**A**) The percentage of HBCs (*n* = 5) for the expression of the investigated 14 markers in relation to the living singlets. (**B**) The visualization of stochastic neighbor embedding (viSNE) analysis of HBCs — 8,500 cells per sample were viSNE-plotted in Cytobank within the living singlets according to the following parameters: iterations: 1,000, perplexity: 30, and theta: 0.5. Blue corresponds to low expression; red corresponds to high expression. (**C**) Comparative heatmap (table’s minimum setting in Cytobank) of mass cytometry data (arcsinh-transformed median intensity values; shown on the color scale) regarding marker expression intensity values of the FlowSOM metaclusters.

Manual gating was used to determine the percentage of HBCs positive for the investigated 14 markers within the living singlets (Figure 7A). The gating strategy is shown in Supplemental Figure 10. In general, HBCs displayed a phenotype similar to immature myeloid cells, characterized by the percentage of CD45^+^ (including low/dim/bright) cells falling between 67% and 83% (mean: 75.9%, SD: 8.9%). The percentage of HBCs positive for other myeloid markers are as follows: CD11b, mean: 13.7%, SD: 10.7%; CD11c, mean: 14.5%, SD: 13.2%; CD14, mean: 23.8%, SD: 9.2%; CD16, mean: 8.9%, SD: 8.3%; HLA-DR, mean: 65.5%, SD: 15.7% (Figure 7A). We found that 81.7% (SD: 9.9%) of HBCs showed positivity for the CD36 fatty acid translocase.

T-distributed stochastic neighbor embedding (t-SNE) analysis deconvoluted the immune landscape of HBCs (Figure 7B). The visualization of t-SNE analysis demonstrated the distribution of single cells in a 14-dimensional mathematical space (*n* = 14, the number of markers under investigation). Plots showing hotspots of CD45 and HLA-DR bright and CD11b, CD11c, CD14, and CD16 myeloid lineage marker molecules represent HBCs with a mature phenotype. However, there were cells showing an uncommitted or immature phenotype within the population that were negative for the investigated markers (blue spots, Figure 7B). The single-cell heterogeneity of HBCs was revealed by unsupervised FlowSOM (self-organizing maps for visualization and interpretation of cytometry data) analysis of CyTOF data (41). FlowSOM generated 5 metaclusters (MCs) representing differential expression patterns of these functional markers, highlighting HBC heterogeneity.

We identified 7 out of 14 markers differentiating the 5 FlowSOM MCs, of which MC1 (CD11b^+^/CD45^+^/CD11c^+^/CD14^lo^/CD16^–^/CD33^+^/HLA-DR^+^) and MC4 (CD11b^lo^/CD45^+^/CD11c^+^/CD14^lo^/CD16^–^/CD33^–^/HLA-DR^+^) represented mature cells, MC3 (CD11b^–^/CD45^lo^/CD11c^–^/CD14^–^/CD16^–^/CD33^–^/HLA-DR^–^) and MC5 (CD11b^–^/CD45^–^/CD11c^–^/CD14^–^/CD16^–^/CD33^–^/HLA-DR^–^) represented immature cells, and MC2 was a transitional cluster (CD11b^–^/CD45^+^/CD11c^–^/CD14^lo^/CD16^–^/CD33^–^/HLA-DR^+^) (Figure 7C). Regarding the HLA-DR expression patterns, our findings are in line with the findings of Yoshida et al. (42).

Thus, based on the CyTOF immunophenotyping, we found that HBCs with relatively low expression of myeloid markers represent a mixture of a mature and immature pool of cells. The plasticity of HBCs with the possible transition among the states identified by CyTOF remains a question and needs further study.

## Discussion

Fetal tissue-resident macrophages play an essential role in several processes during embryogenesis, including angiogenesis, neurogenesis, bone formation, and hematopoiesis, and provide a source of cellular proliferation for tissue-resident macrophages throughout the body (43, 44). Considering the literature and recent findings on HBCs’ unique origin, gestational adaptations, and the placenta’s significance, this cell type emerges as an intriguing research topic in biology and medicine; however, molecular mechanisms controlling HBCs have yet to be elucidated. The primary aim of this study was to isolate pure human HBCs, free from contamination by maternal or non-macrophage fetal cells, to enable access to a clean, HBC-specific transcriptional profile. We used bulk RNA-seq to assess emerging gene expression patterns and to maximize sensitivity for low-abundance transcripts, such as transcription factors, which are often underrepresented in single-cell data. Thus, this approach also complemented our bulk ATAC-seq analysis, allowing us to link gene expression with chromatin accessibility and identify candidate regulatory factors with greater confidence. Comparing term HBC transcriptomes to other fetal and adult human macrophages helped to contextualize HBC gene expression patterns and shed light on substantial differences with possible functional significance. The regulation of angiogenesis, placental lipid metabolism, and transplacental lipid transport emerge as key functions of term HBCs. Gene expression data raise the question of whether HBCs have potent antimicrobial properties. Through our in vitro induction experiments, we could gain insight into the gene expression regulation of CD36. In order to bridge the gap between the bulk nature of our approach, we implemented CyTOF analysis to study cellular heterogeneity on a proteomic level and identified myeloid phenotypes and heterogenous maturation states in our isolates. This approach provides a foundation for basic biology, disease modeling, and translational applications by identifying distinct gene expression patterns (including DEGs and HBC-specific transcripts).

### The likely functions of HBCs based on gene expression.

Based on our results and analysis, HBCs have a distinct gene expression pattern different from other fetal and adult macrophage subpopulations. In assessing the function of highly or exclusively expressed individual genes and emerging gene expression patterns, we found that HBCs play a role in maintaining placental structural and functional integrity (high ranking of extracellular matrix genes on Figure 3A and Supplemental Figure 5B), development of placental microarchitecture (high ranking of angiogenesis genes on Figure 3A and Supplemental Figure 5A), lipid metabolism and lipid signaling (high ranking of NR genes on Figure 3, A and B). The low ranking of microorganism genes suggest that third-trimester HBCs have inferior antimicrobial properties within this comparative context (Figure 3A and Supplemental Figure 6C). This finding resonates with the findings of Yoshida et al., who found that term HBCs have diminished activity against *L*. *monocytogenes* infection compared with first-trimester HBCs (42).

Dissecting the list of DEGs highly expressed in HBCs can serve as a basis for subsequent experiments characterizing cell type functions using mechanistic approaches. High expression of *SEPP1* suggests HBCs’ role in maintaining placental oxidative balance and transplacental selenium transport, while *TGFB1* emerges as a cytokine with a substantial role in HBC-trophoblast communication or maintaining immunological balance at the fetal-maternal interface. Furthermore, many complement components and regulatory factors, including *C1QA*, *C1QC*, and *VSIG4*, are exceptionally highly expressed genes located in C1. Previous literature suggests the significance of placental complement cascade dysregulation in the pathophysiology of villitis of unknown etiology (45, 46) and in the pathogenesis of preeclampsia (47). Supporting the relevance of this pathway, studies have shown that C1 complex–deficient mice exhibit reduced litter sizes, and that anti-C1q antibodies may contribute to recurrent pregnancy loss in individuals with lupus nephritis and antiphospholipid syndrome (48, 49). The enrichment of complement components in term HBCs was also highlighted by Yoshida et al. (42).

### Identification of specific and selective HBC genes.

Gene expression data alone are rarely sufficient to draw definitive conclusions about a gene’s function. However, comparing the expression of specific genes across different cell types can generate stronger, testable hypotheses about the roles of those cell types (Figure 2E). Within this comparative context, we identified several HBC-specific genes known to play a role in gestational physiology that are associated with various diseases, including pregnancy complications. Examples include *TAC3* associated with infertility (50), *CSH1* with choriocarcinoma (51), and *PAPPA2* with pre-eclampsia (52).

*H19* and *IGF2* form an imprinted cluster on chromosome 11, whose lncRNA and growth factor products reciprocally regulate fetal and placental development (53, 54). *H19* is expressed from the maternal allele and limits growth, in part by repressing *IGF2*, which when expressed from the paternal allele promotes placental nutrient transport, hormone secretion, and trophoblast differentiation/invasion. Their activity is modulated by IGF-binding proteins, notably *IGFBP3* and *IGFBP4* (C1 HBC–specific genes), and misregulation underlies Silver-Russell and Beckwith-Wiedemann syndromes, both of which are growth disorders (55–57). NOTUM, a secreted WNT deacetylase, restrains WNT-driven trophoblast stem cell maintenance to permit extravillous differentiation, while APOLD1 organizes endothelial junctions and cytoskeletal architecture to regulate barrier permeability (58, 59).

### The epigenomic underpinning of HBC gene expression.

Comparing ATAC-seq data from HBCs with ATAC-seq data from healthy human AMs and MDMs revealed insights into HBC gene expression regulation. Regarding promoter regions, we hypothesize that enrichment of the RFX binding motif and gene expression of RFX family member genes may indicate a transcription factor with a potential role specific to either the cell type or the gestational phase in HBCs. RFX factors that share a common DNA-binding motif are indispensable for MHC II expression; mutations in RFX genes cause bare lymphocyte syndrome, leading to drastically reduced MHC II expression on immune cells (60, 61). The high expression of RFX5 and the enrichment of its binding motif aligns with previous findings on the transition between first-trimester HLA-DR^–^ HBCs and third-trimester HLA-DR^+^ HBCs, as well as with our CyTOF analysis (Figure 7) (42). Further dissection of the RFX transcription factor family’s functional relevance requires a mechanistic approach, possibly involving animal studies, epigenetic analysis, or gene knockdown experiments.

The enrichment of transcription factor binding motifs characteristic of myeloid cells, as well as motifs binding NR4A members and estrogen receptors in the HBC-specific enhancer cluster resonate with gene expression data that highlighted the significance of NR signaling (Figure 3A) with high expression of certain NRs and GR-regulated genes. If FoxA1 is capable of binding to the motif, it may also serve as a pioneer transcription factor for ERα, which could bind the IR3 NR motif (enriched by 7.07%) within HBCs (Figure 4C). These findings lay a foundation for future projects focusing on cell identity and differentiation, more extensive mechanistic experiments to study HBC responsiveness, and potential therapeutic targets.

### HBCs as lipid-rich and lipid-handling macrophages.

Cellular morphology; enrichment of NR binding sites; and high expression of certain NRs, *CD36*, *PLTP*, *SREBP1*, ATP binding cassette subfamily A member 1 (*ABCA1*), and ATP binding cassette subfamily G member 1 (*ABCG1*), all converge in the same direction, highlighting fatty acid and cholesterol functionality in HBCs (Figure 2D). CD36, a key scavenger and lipid uptake receptor under the control of PPARγ, CEBPα, and NR1D2, has several ligands including free fatty acids, lipoproteins (e.g., LDL), collagen, and thrombospondin (62, 63). CD36 is present in several tissues and cell types but has an unclear role and a vague pathological significance in the placenta. Dubé et al. found an association between maternal obesity and increased newborn cholesterol and LDL levels, as well as increased placental *CD36* expression at both mRNA and protein levels (64). CD36 is crucial in forming foamy macrophages in atherosclerotic lesions, morphologically similar to HBCs on microscopic Cytospin images. While CD36 is well-characterized as a scavenger receptor in other macrophage populations, its specific function in HBCs remains undefined. Its expression suggests a potential role in lipid uptake and clearance of cellular debris or microbial antigens, but direct functional evidence in the placental context is currently lacking. Performing CyTOF analysis allowed us to examine CD36 expression in the HBC isolates on the protein level. The consistently high expression of CD36 protein in all subgroups (Figure 7, A–C) backs the gene expression data and supports the proposed significance. Notably, our clinical inclusion/exclusion criteria may not exclude the influence of maternal metabolic imbalances on placental microenvironment, as we did not consider maternal obesity or laboratory parameters indicative of metabolic status. However, as part of physiological changes in pregnancy, maternal blood levels show a natural increase (65). In this context, the significant increase in leptin mRNA expression in pre-eclamptic placentas contributes to altered immune and metabolic functions and may support the existence of a pathway by which HBCs may be linked to the pathogenesis of this pregnancy complication of high morbidity (66). Whether this is a correlative or causative relationship remains to be determined.

Our IL-4– and rosiglitazone-mediated PPARγ induction experiments, although limited in scope, confirmed the interaction of the pathways and raised the possibility of synergistic interactions (e.g., *CD36*, *ANGPL4*, *IL1RN*), which need to be verified using dose-response studies (Figure 6B). These experiments further underpin the adaptability of HBCs and pave the way for testing additional ligands to alter gene expression, intercellular interactions, and ultimately the functional or pathophysiological role of HBCs.

All HBC isolates analyzed in this study were derived from healthy term placentas delivered via cesarean section. While our data establish a foundational “parts list” of gene expression in normal HBCs, future studies are needed to investigate how gene expression and regulatory programs may vary with pregnancy complications or gestational age. Our findings support the hypothesis that HBCs possess distinct transcriptional and regulatory profiles; however, they also raise new questions that extend beyond the scope of this work.

An important area for future investigation is the interaction between HBCs and neighboring cells, including trophoblasts and endothelial and mesenchymal cells. Given species-specific differences in placental structure and physiology, addressing this question may require advanced models such as organoids, cocultures, micro-physiological systems, in vivo imaging, and in silico tools like CellChat. Furthermore, given the specificities of each trimester during pregnancy, the function of HBCs may show dynamic changes and adaptations at different times and, remarkably, ready programmability even at term (67).

This exploratory study provides a foundational multiomic characterization of term HBCs. We acknowledge that a limitation of this study is the relatively small sample size (*n* = 5 donors), which could lower our power to capture biological variation in this system. Nonetheless, donor-to-donor correlation was high, as observed in our gene expression (Pearson’s correlation ≥ 0.77) and chromatin accessibility assays (Pearson’s correlation ≥ 0.88), indicating that our donors showed high overall transcriptomic and epigenomic similarity. In spite of the homogeneity among the donors, we showed that consistent molecular features differentiate HBCs from other fetal and adult macrophage populations, irrespective of the assay used (CyTOF: 5 donors; qPCR/RNA-seq: 4 donors; ATAC-seq: 3 donors). Importantly, this level of correlation is also consistent with intrapopulation variation estimates from cellular atlases such as the Tabula Sapiens, where gene expression among resident macrophages in the same tissue was highly correlated (68). It should be noted that acquiring human placental samples for research is a challenging task (given the ethical concerns and strict inclusion and exclusion criteria, etc.), and this study represents an important step toward the exploration of this unique and specialized immune cell population. As with any study using human samples, future studies are always warranted to include larger cohorts to capture additional variation. While we identified transcriptional and chromatin programs suggesting regulatory roles for NRs such as PPARγ and NR4A family members, functional validation of these pathways in vivo remains to be done. Finally, although in vitro stimulation assays indicated transcriptional plasticity, we did not perform additional functional assays, such as lipid uptake, phagocytic, or coculture experiments in this study; these assays can be included in future studies. Ongoing work to develop in vitro placental models and functional assays will be essential to validate and expand upon these findings.

Overall, these results will lay the foundation for further studies focusing on the isolation and characterization of HBCs from placentas from complicated pregnancies, applying single-cell approaches to uncover the degree and characteristics of heterogeneity, and in vitro analyses of programmability and cell-cell interactions (i.e., trophoblasts, endothelial cells) using cocultures and microrheological systems.

## Methods

### Sex as a biological variable.

The study involved 3 male and 2 female newborns. Sex was not considered as a biological variable, and findings are expected to apply to both sexes.

### Participants involved in the study.

Participant selection was based on inclusion and exclusion criteria as shown in Supplemental Table 2. An overview of donor samples and associated technical replicates used across the experiments and analyses is provided in Supplemental Table 3.

### Tissue processing and cell isolation.

To gain HBCs of an appropriate number and purity, we adapted the protocol of Tang et al. (68). Briefly, the placentas were transferred to a BSL-2 laboratory within 20 minutes of delivery. The fetal membranes were first separated, and the villous tissue was obtained by dissecting the placenta. The tissue was minced and rinsed with calcium- and magnesium-free PBS. The chopped tissue was digested in 3 cycles with 0.25% trypsin (Thermo Fisher Scientific)/0.08 mg/mL DNase I (Roche) digestion solution (37°C water bath with orbital shaker for 15, 30, and 45 minutes). The undigested tissue fragments (containing HBCs) were digested in 1 mg/mL collagenase A (Roche)/0.1 mg/mL DNase I digestion solution (37°C water bath with orbital shaker for 45 minutes) followed by filtration through a 100 μm sieve. The pooled and filtered cell suspensions were centrifuged before separation of HBCs by Percoll (GE) gradients; 7.5 mL of 40%, 35%, 30%, and 20% Percoll gradients were used, and 5 mL of cell suspension was layered on top of the gradients. The suspension-loaded gradients were centrifuged for 20 minutes at 1,200*g* at room temperature (RT) without brake. The HBCs were found at the interfaces between 35% and 30% layers and between 30% and 20% layers. After gradient-based cell separation, the cell suspension was further purified by incubating with magnetic beads (Invitrogen) conjugated to anti-EGFR (sc-120, Santa Cruz Biotechnology) and anti-CD10 (312202, BioLegend) antibodies, using 25 μL of beads per 10 million cells. The cell suspension was collected, and the beads were removed by placing the suspension into a paramagnetic field. The total cell number was then counted.

### Flow cytometry analysis and cell sorting.

The EGFR^–^ CD10^–^cells were labeled for anti-hCD163-APC (333609, BioLegend) antibody. The FcR blocking reagent (Miltenyi Biotec) was used to prevent the nonspecific binding of antibody conjugates. To discriminate live and dead cells, eBioscience Fixable Viability Dye eFluor 506 (Thermo Fischer Scientific) was used based on the manufacturer’s recommendation. The CD163^+^ HBCs were sorted by FACSAria III (BD Biosciences) based on cell size, singularity, viability, and CD163 positivity, and HBCs were used for Cytospin, RNA-seq, and ATAC-seq analysis.

### Quality control of the applied HBC isolation process using single-cell RNA-seq.

Initially, quality control filtering was applied to the single-cell data, aiming to retain only viable cells. Cells falling within the extreme 0.5th percentile of both the number of identified features and total count distributions were excluded. Additionally, cells in which mitochondrial counts constituted more than 15% of the total counts were filtered out (Supplemental Figure 2, A and B). The quality control approach maintained the nFeature/nCounts ratio at 0.9, indicating a strong correlation between these 2 features and suggesting a minimal presence of doublets.

The single-cell analysis allowed us to perform unsupervised cell type annotation through the SingleR package. By comparing our cells’ transcriptional profile to those from the Human Primary Cell Atlas database (64), the software identified 2 major cell identities (Supplemental Figure 2C). The largest population, comprising 89.2% of total cells, had a clear macrophage/monocytic identity, being automatically annotated as such. The second, 9.8% of the cells, had a mixed profile ranging from tissue stem cells to fibroblasts.

### Determination of maternal contamination.

To assess potential maternal cellular contamination, we developed a protocol predicting contaminating nonfetal cell proportions based on sex-associated gene expression differences. Using a single-cell transcriptomics library from a female/female sample, we derived a pseudo-bulk RNA to estimate XIST expression (69). Comparing XIST expression percentages in male offsprings’ bulk RNA female/male to female/male samples (percentage in female/male sample / percentage in female/female sample × 100) revealed insights into maternal contamination, given the child’s sex. To further complement quality control, we performed principal component analysis comparing HBCs with 2 subpopulations of decidual macrophages (dM1, dM2), maternal placental macrophages (PM3), first-trimester Hofbauer cells (HBP_FT), and third-trimester Hofbauer cells (HBP_TT). Reference single-cell RNA-seq data (dM1, dM2, PM3, HBP_FT) were obtained from the publication of Vento-Tormo et al. (13) and HBP_TT from Sureshchandra et al. (16). Following standard quality control (doublet removal, feature/count thresholds), cells were subset based on original annotations and stratified by biological replicate. Samples with fewer than 100 cells per cell type and biological replicate were excluded. Remaining cells per replicate were aggregated into pseudo-bulk profiles.

To integrate pseudo-bulk data with our bulk RNA-seq HBC dataset, both were normalized for library size and merged by intersecting gene sets. Batch effects between pseudo-bulk and bulk data were corrected using surrogate variable analysis prior to principal component analysis.

### Collection of public RNA-seq and ATAC-seq data.

Publicly available fetal and adult RNA-seq and adult ATAC-seq data were collected from the Sequence Read Archive (SRA) database, and the raw sequences were downloaded from the European Nucleotide Archive (ENA) database of the EMBL-EBI. The list of the used data and their identifiers is available in Supplemental Table 4. The raw values are provided in Supplemental Table 5 and Supplemental Table 6.

### RNA-seq analysis.

RNA-seq analysis was carried out on *n* = 4 placentas. Out of 3 placentas, 2 technical replicates were involved to assess variability and strengthen quality control and for technical consistency.

Raw sequence reads of all RNA-seq data were uniformly aligned to the hg19 reference genome assembly with HISAT2 (v2.1.0) using default parameters (70). From the BAM files, transcripts were assembled with StringTie (v1.3.4d) (71). BAM files were indexed with SAMtools (v1.7), and then coverage (bedgraph) files were generated by HOMER’s makeUCSCfile program (72, 73). Gene expression levels were determined in FPKM values.

For secondary analyses, genes that showed a higher than 0 FPKM value in at least 1 sample were considered expressed and included in the comparative analyses. Then, for each sample, the FPKM value was normalized by the decile value of the given sample. The differentially expressed genes were determined with 1-way ANOVA supplemented with a post hoc Tukey’s honestly significant difference (HSD) statistical test using the stats, tidyverse, and sqldf packages in R. The applied cutoff values (0.05 as *P* value and 2 as fold difference) are indicated in the figure descriptions in all cases. The multidimensional scaling (MDS) plot was generated by the plotMDS function of the limma package, the sample-to-sample distance plot was visualized by the edgeR and pheatmap packages, and the Pearson’s correlation-based heatmap was generated using the pheatmap package in R (v3.5.1) (74). Only bulk RNA-seq datasets were compared with our own bulk RNA-seq result and are included in Figures 2–5 and Supplemental Figures 4–6. The biological pathways were predicted with Metascape (v3.5.20230501) (75). K-means clustering of the genes was carried out by Cluster 3.0, and the applied similarity metric was the centered correlation (76).

Genes for the sensing repertoire of tissue-resident macrophages were collected from the original article and were supplemented with the members of the gene or receptor (super)families and lectins (30, 77). Genes with an average normalized expression lower than 0.5 FPKM in all tissue types were not shown. Gene sets for the functional analysis were derived from the Panther database. Normalized expression values for the consensus gene set, the genes associated with the clusters, and those encoding macrophage-specific transcription factors are listed in the Supporting Data Values file.

### In vitro induction.

Primary HBCs (from *n* = 4 placentas) after isolation and sorting were plated onto multi-well culture plates (approximately 1 million cells per well) in RPMI supplemented with 5% FBS, 1% penicillin/streptomycin, and 25 mM HEPES. We treated freshly plated HBCs with IL-4 (20 ng/μL) and/or the synthetic agonist of PPARγ, rosiglitazone (1 μM). Cells were harvested 24 hours after cytokine and ligand treatment. Cotreatment happened simultaneously. Total RNA was isolated from cells using Tri Reagent (MRC) according to the manufacturer’s protocol. RNA was transcribed into cDNA; transcript quantification was performed by quantitative real-time PCR (RT-PCR) using user-designed TaqMan assays or TaqMan Gene Expression Assays. Gene expression was quantified by the comparative threshold cycle method and normalized to cyclophilin A expression (78). The raw values are provided in Supplemental Table 7.

### ATAC-seq analysis.

ATAC-seq analysis was carried out on *n* = 3 placentas. Out of 2 placentas, 2 technical replicates were involved to assess variability and strengthen quality control and for technical consistency.

Raw sequence reads were aligned to the hg19 reference genome assembly with default parameters by using the BWA tool (v07.17); single-end reads were aligned by setting the *samse*, and paired-end reads were aligned by using the *sampe* parameter (79). BAM files were generated with SAMtools (v1.7) (70). Narrow peaks were predicted with the callpeak function of MACS2, and their widths were fixed to 200 bp relative to their summits (80). Artifacts were removed according to the blacklisted genomic regions of ENCODE (81). Genome coverage (bedgraph) files were generated by HOMER’s makeUCSCfile, and then they were indexed to tdf files with igvtools (v2.3.98) (82). The fragment length was set to 150 bp.

For the secondary analyses, a consensus peak set was generated by using the mergeBed command of bedtools (v2.27.1) (83). Peaks that could be predicted from at least 5 samples were part of the consensus set. Then, for each sample, reads per kilobase per million mapped reads (RPKM) values were calculated on the consensus peak set by the coverageBed command of bedtools, and the RPKM values were normalized by the decile value of the given sample. The Pearson’s correlation-based heatmap was generated using the pheatmap package in R (v3.5.1).

By using the decile-normalized values, the differentially accessible chromatin regions were defined by 1-way ANOVA supplemented with a post hoc Tukey’s HSD statistical test. As the cutoffs, a *P* value of 0.05 or less and fold difference of 2 or greater were applied. K-means clustering of the genomic regions was carried out by Cluster 3.0, and the applied similarity metric was the centered correlation (76). The consensus peak set, the differentially accessible chromatin regions, and the regions associated with the clusters are listed with their normalized RPKM values in the Supporting Data Values file.

### De novo motif enrichment analysis.

First, the separation of the TSS-proximal (promoter-TSS and 5′ UTR) and TSS-distal (introns, intergenic, exons, 3′ UTR, and TTS) regions was determined by HOMER’s annotatePeaks. Then, de novo motif enrichment analysis was carried out by HOMER’s findMotifsGenome.pl program and was performed on the summit ± 100 bp regions of all the TSS-proximal (promoter) and the summit ± 100 bp of the top 1,000 TSS-distal (enhancer) regions (72). The targeted motif lengths were 10, 12, 14, and 16 bp. *P* values were calculated by comparing the enrichment within the target regions with that of a random set of regions (background) generated by HOMER.

### Visualization.

Row-normalized gene expression heatmaps were visualized in R by the pheatmap package. Heatmaps representing absolute gene expression values were visualized by JavaTreeView (v1.1.6r4) (84). Bar charts and dot plots were plotted by using GraphPad Prism v.9. Genome coverage (bedgraph) files were visualized by Integrative Genomics Viewer (v2.4.16), where the replicates that belong together were depicted as overlay tracks (82). Figure 6A was created in BioRender (https://BioRender.com).

### Mass cytometry.

All 5 placentas were involved for mass cytometry analysis. Single-cell mass cytometry was performed as described previously by our group with minor modifications (85, 86). Briefly, cryotubes were thawed in a 37°C water bath for 2 minutes, and cells were transferred into 14 mL RPMI (Capricorn) at 37°C and centrifuged at 350*g* for 6 minutes (washed) at RT (24°C). Cells were washed once more with 10 mL RPMI, cells were counted using a Bürker-chamber, and viability was determined by Trypan blue (Merck) exclusion. Cells were washed with 2 mL Maxpar cell staining buffer (MCSB; Fluidigm), resuspended in 500 μL MCSB-containing cell ID ^195^Pt-cisplatin (Standard BioTools) (stock 5 mM, 1,000× diluted), and incubated for 3 minutes on ice. Cells were washed with 2 mL MCSB 2 times and resuspended in 50 μL MCSB supplemented with 1:20 v/v TruStain FcX FC receptor blocking solution (BioLegend) and incubated at RT for 10 minutes. Cells were stained with the Human Monocyte-Macrophage Phenotyping Panel Kit (Standard BioTools) according to the instructions of the manufacturer in the final volume of 100 μL MCSB and incubated at RT for 30 minutes. The antibodies used for CyTOF are listed in Supplemental Table 8. Cells were washed twice with MCSB, prefixed with 500 μL of Pierce formaldehyde (stock w/v 16%) (Thermo Fisher Scientific) solution diluted in PBS to 1.6%, and incubated at RT for 10 minutes. Stained and prefixed cells were centrifuged at 800*g* at RT for 6 minutes and resuspended in 800 μL Fix & Perm solution (Standard BioTools) supplemented with 1:1,000 [v/v] ^191^Ir-^193^Ir DNA intercalator (Standard BioTools) for overnight incubation. Samples were washed 2 times with MCSB and once with PBS, and then filtered through a 30 μm Celltrics gravity filter (Sysmex). The cell concentration was adjusted to 7 × 10^5^/mL in Cell Acquisition Solution (Standard BioTools) including EQ 4-element calibration beads (Standard BioTools) at a 1:10 ratio [v/v]. Applying the WB injector (Standard BioTools), cells were acquired using a properly tuned Helios mass cytometer (Standard BioTools). The generated flow cytometry standard (FCS) files were randomized and normalized with the default settings of the internal FCS-processing unit of the CyTOF software (Fluidigm, version:7.0.8493). The randomized and normalized FCS files were uploaded to the Cytobank analysis platform (Beckman Coulter; https://premium.cytobank.org/cytobank/experiments/494993).

Exclusion of normalized beads, dead cells, debris, doublets, and manual gating was performed as presented in Supplemental Figure 10. The percentage of the populations defined by manual gating was plotted in GraphPad Prism 8 (Dotmatics). The anti-CD7 signal with close intensity to the background was excluded from subsequent analysis. In total, 300,000 single cells and 14 markers were used to create a t-SNE map of Hofbauer cells. High-dimensional reduction and visualization were performed using the t-SNE algorithm. For the unsupervised definition of main subpopulations, we performed a self-organizing map-based FlowSOM metaclustering analysis. The CyTOF marker intensities were inverse-hyperbolic sine-transformed (arcsinh) with cofactor 5 in Cytobank. We identified 5 different metaclusters that were separately subclustered in another round of FlowSOM.

The FlowSOM was performed in Cytobank Premium. The expression intensities were expressed as arcsinh values that were calculated by applying the arcsinh equation divided by the scale argument to the measured metal intensity value. The marker expression intensities of the acquired HBCs in CyTOF were beyond the values of peripheral myeloid cells using the commercially available Human Monocyte-Macrophage Phenotyping Panel Kit for CyTOF. Reference PBMC samples were acquired simultaneously with the HBCs as an internal control (data not shown).

### Statistics.

For RNA-seq analysis, differentially expressed genes were identified by 1-way ANOVA with post hoc Tukey’s HSD (using the stats, tidyverse, and sqldf packages in R). Genes with a *P* value of 0.05 or less and a fold difference of 2 or greater were considered significant. Scatterplots indicate mean (line) and SD (whiskers).

For ATAC-seq analysis, differentially accessible chromatin regions were defined by 1-way ANOVA with post hoc Tukey’s HSD, applying the same thresholds (*P* ≤ 0.05 and fold difference ≥ 2). K-means clustering of genomic regions used centered correlation as the similarity metric. De novo motif enrichment was assessed in HOMER by comparing the target regions to a random set of regions (background), with significance determined by *P* less than or equal to 0.05.

### Study approval.

All the procedures were approved by the Ethics Committee of the University of Debrecen (protocol DE RKEB/IKEB 6426-2023) and the Policy Administration Services of Public Health of the Government Office (protocol BM/1425-3/2024). Informed written consent was obtained from all the participants involved in this research, and the study was performed in agreement with the ethical standards of the Declaration of Helsinki.

### Data availability.

The generated next-generation sequencing datasets are available in NCBI’s Gene Expression Omnibus under GSE255954 (ATAC-seq) and GSE255955 (RNA-seq).

## Author contributions

BRB, TD, and LN planned the experimental project, DB planned the bioinformatic approaches, and they drafted the manuscript. BRB, KB, and TC isolated HBCs from placentas and produced the RNA-seq samples. PT carried out the ATAC-seq experiments. DB, GN, and JCRF performed the computational analyses. KB and NCS performed the flow cytometry measurements. Patient recruitment was done by TD and ZTK. MT carried out Cytospin slide preparation. CyTOF was performed and analyzed by PN and GJS. RT-qPCR was executed by BRB, KB, and TC. The HBC isolation protocol was created and adapted with the help of ZT and SG. BRB, DB, GJS, JCRF, KB, and PN designed the figures. LN directed and supervised the work. All authors discussed the results and commented on the manuscript.

The order of co–first authorship was determined by weighing relative contributions to experimental design, execution, analyses, conceptualization, and writing of the manuscript.

## Funding support

- BRB by the EKÖP-25-3 university research scholarship program of the Ministry of Culture and Innovation from the National Research, Development, and Innovation Fund, Hungary.
- National Research, Development, and Innovation Office, Hungary (OTKA K147147, ADVANCED-152422 to the Nuclear Receptor Research Lab and FK142877 to GJS, FK146945 to GN, and PD137902 to DB).
- János Bolyai Research Scholarship of the Hungarian Academy of Sciences (BO/00582/22/8 to GJS).
- NCS by the European Union’s Horizon 2020 research and innovation program under the Marie Skłodowska-Curie grant agreement 860034.

## Supplementary Material

Supplemental data

Supplemental table 4

Supplemental table 5

Supplemental table 6

Supplemental table 7

Supporting data values

## Figures and Tables

**Table 1:**
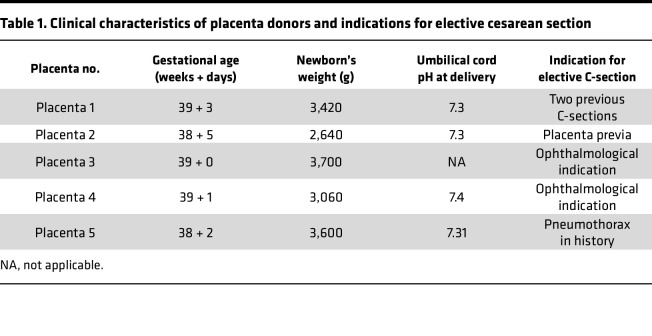
Clinical characteristics of placenta donors and indications for elective cesarean section

**Table 2:**
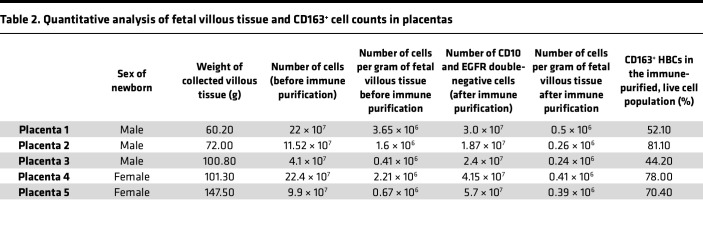
Quantitative analysis of fetal villous tissue and CD163^+^ cell counts in placentas

## Version 1 12/23/2025

In-Press Preview

## Version 2 02/09/2026

Electronic publication
