# Supplementary material for: Distinct transcriptional and epigenomic programs define Hofbauer cells in term placenta
Source: JCI Insight. 2025 Dec 23;11(3):e195801. doi: 10.1172/jci.insight.195801 (PMC12892902; doi:10.1172/jci.insight.195801)
Supplement: Supplemental data [file jciinsight-11-195801-s032.pdf]

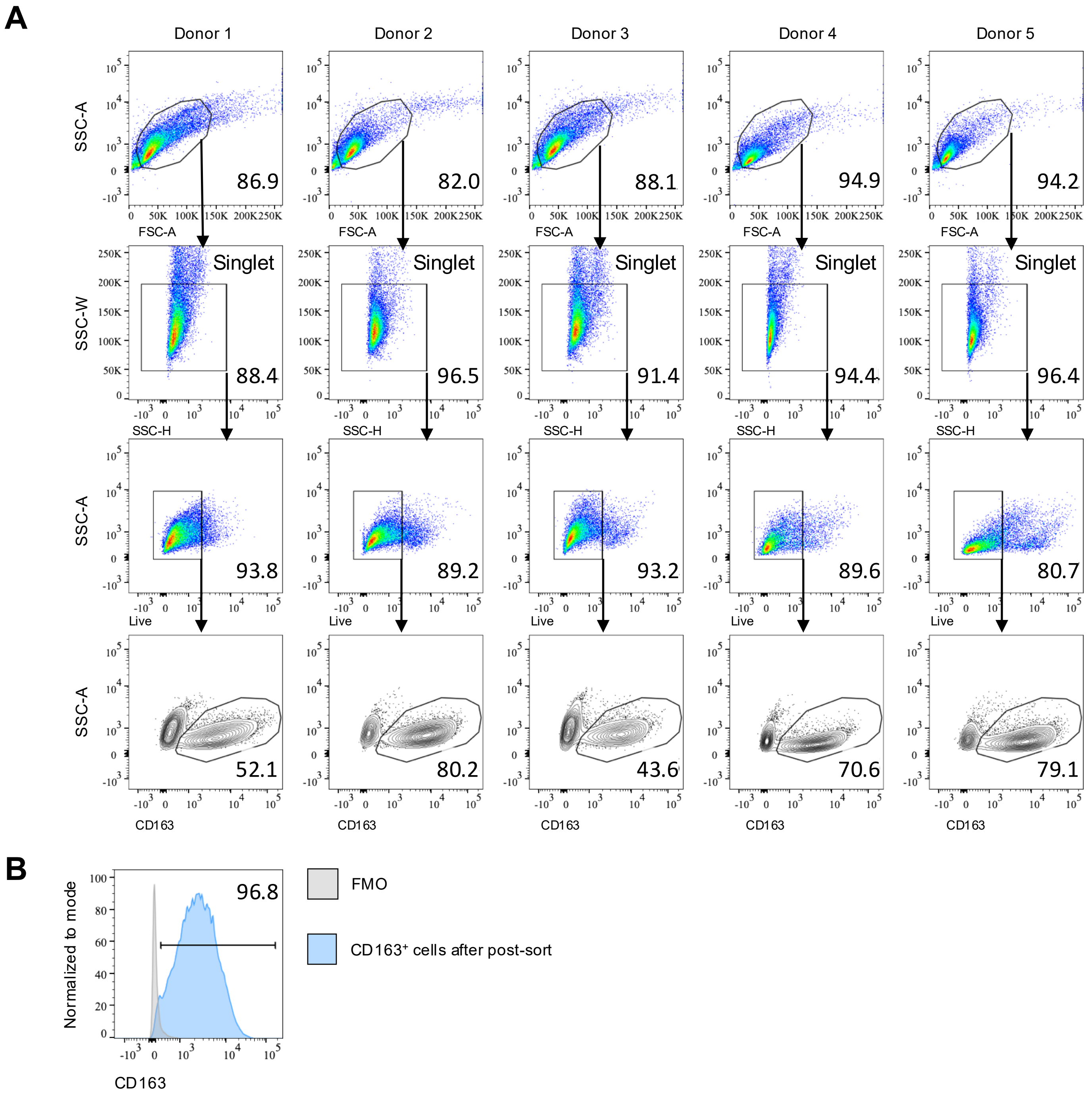

Supplementary Figure 1. Flow cytometry strategy and purity assessment of CD163^{+} HBCs. **(A)** The gating strategy to assess and sort Hofbauer cells by flow cytometry. **(B)** Histogram overlay shows the purity of cells after CD163-based cell sorting. The data are derived from one representative donor.

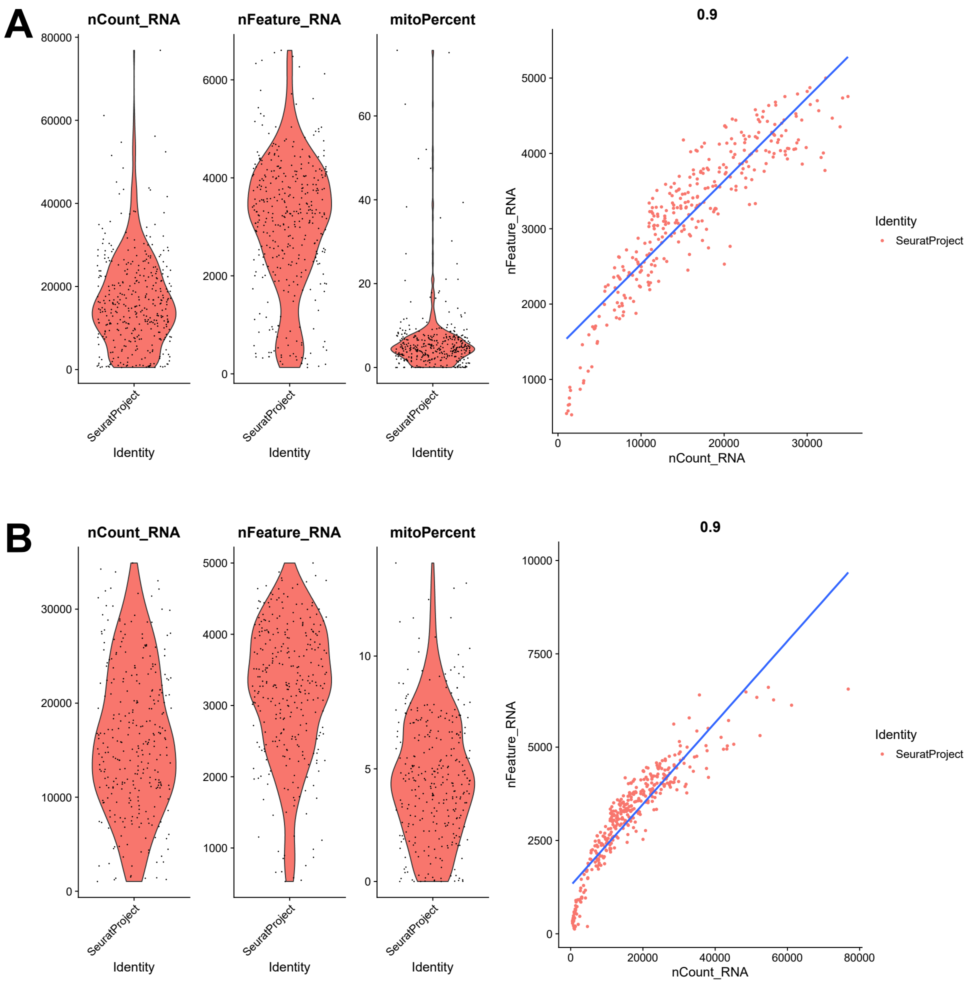

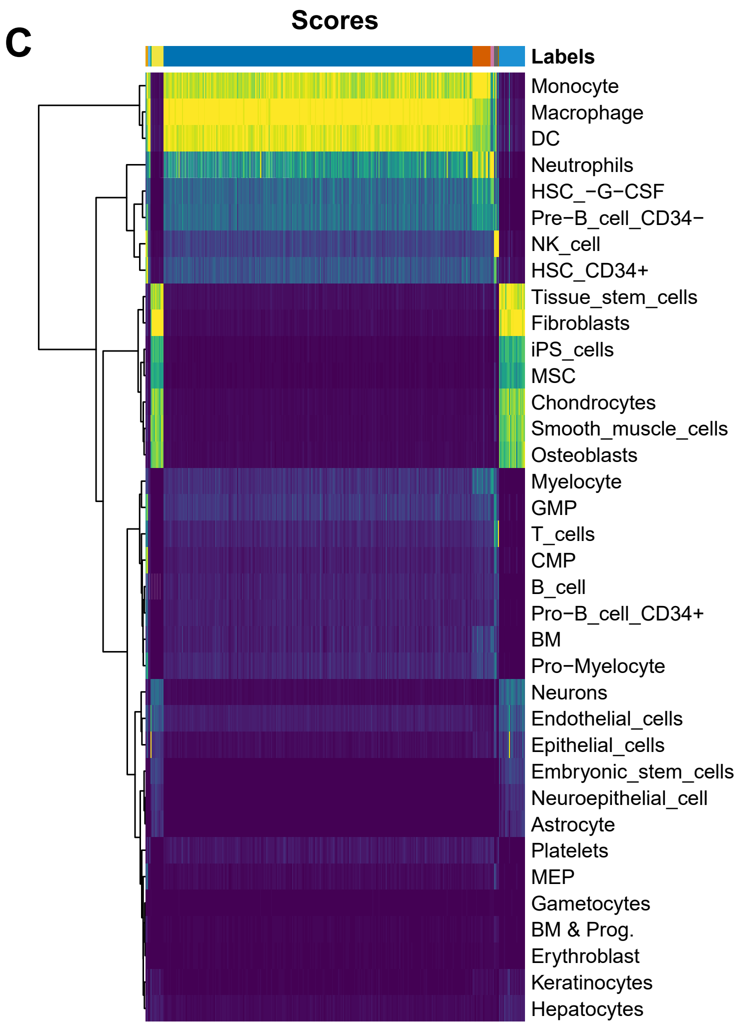

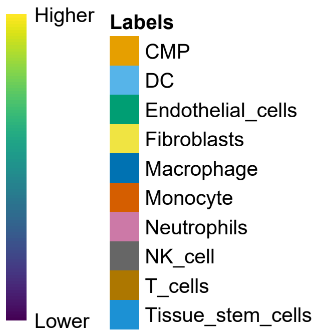

Supplementary Figure 2. Single-cell RNA-seq quality control and cell type annotation. **(A)** Violin plots illustrating the distribution of nCount, nFeature, and mitochondrial RNA percentage (mitoPercent) across single cells before filtering, alongside a scatter plot depicting the correlation between nFeature and nCount. The upper value represents the correlation coefficient, with the blue line indicating the correlation trend, where a value of 1 signifies perfect correlation. **(B)** The same plots as in (A), but after quality control filtering. **(C)** Confidence scores for SingleR unsupervised cell type annotation. Each cell's transcriptional profile was compared to previously annotated cells from the Human Single Cell Atlas (64), and the confidence of each inferred annotation is visualized as a heatmap.

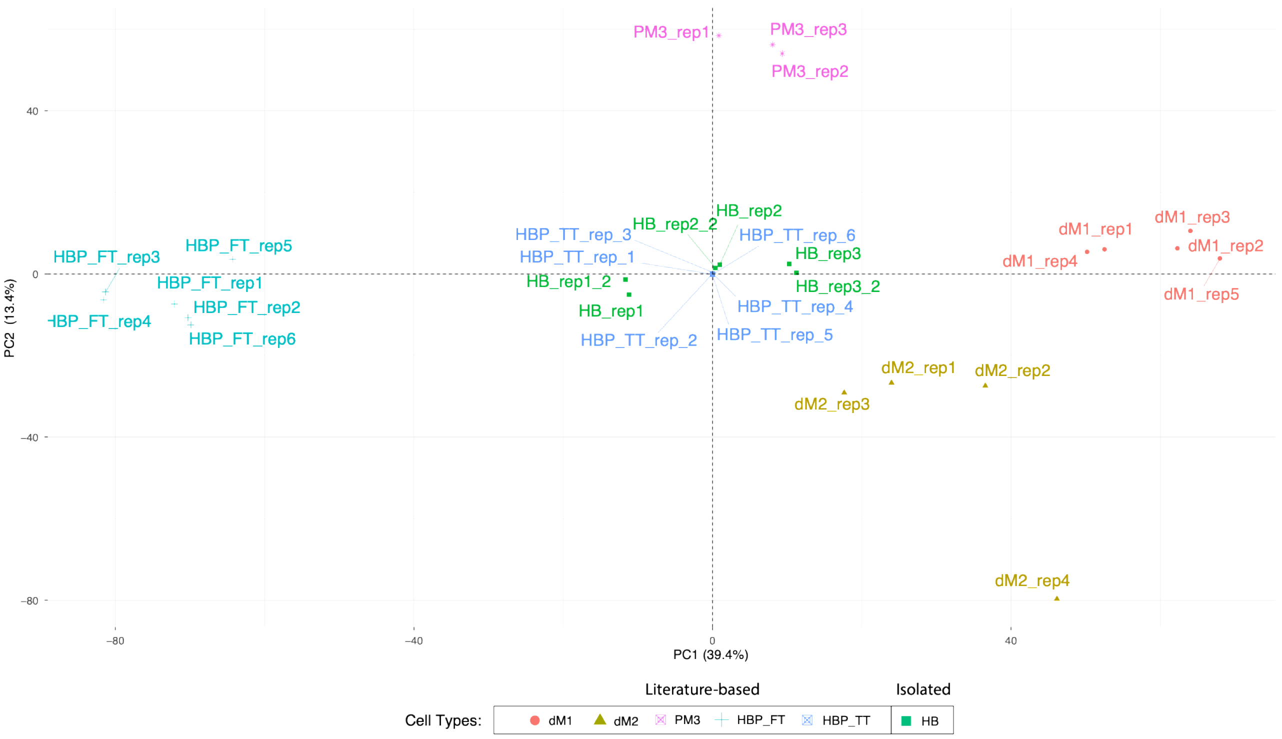

Supplementary Figure 3. Principal component analysis of term Hofbauer cells versus decidual and placental macrophage references

Our HB bulk RNA-seq replicates (green squares) are compared with pseudobulk references from scRNA-seq [Vento-Tormo *et al.* (13)]: dM1 (red circles), dM2 (yellow triangles), PM3 (magenta squares), first-trimester HBCs (blue crosses) and third trimester HBCs (Sureshchandra S, Doratt BM, True H, et al. Multimodal profiling of term human decidua demonstrates immune adaptations with pregravid obesity. Cell Rep. 2023;42(7):112769. doi:10.1016/j.celrep.2023.112769. scRNA-seq references were QC'd and aggregated to pseudobulk by replicate; datasets were library-size normalized, merged on intersecting genes, and SVA-corrected before PCA. Each point is a biological replicate; dashed lines mark zero axes. Abbreviations: HB, term Hofbauer cells; HBP, first-trimester Hofbauer cells; PM3, maternal placental macrophages; dM1/dM2, decidual macrophage subsets; PCA, principal component analysis; SVA, surrogate variable analysis.

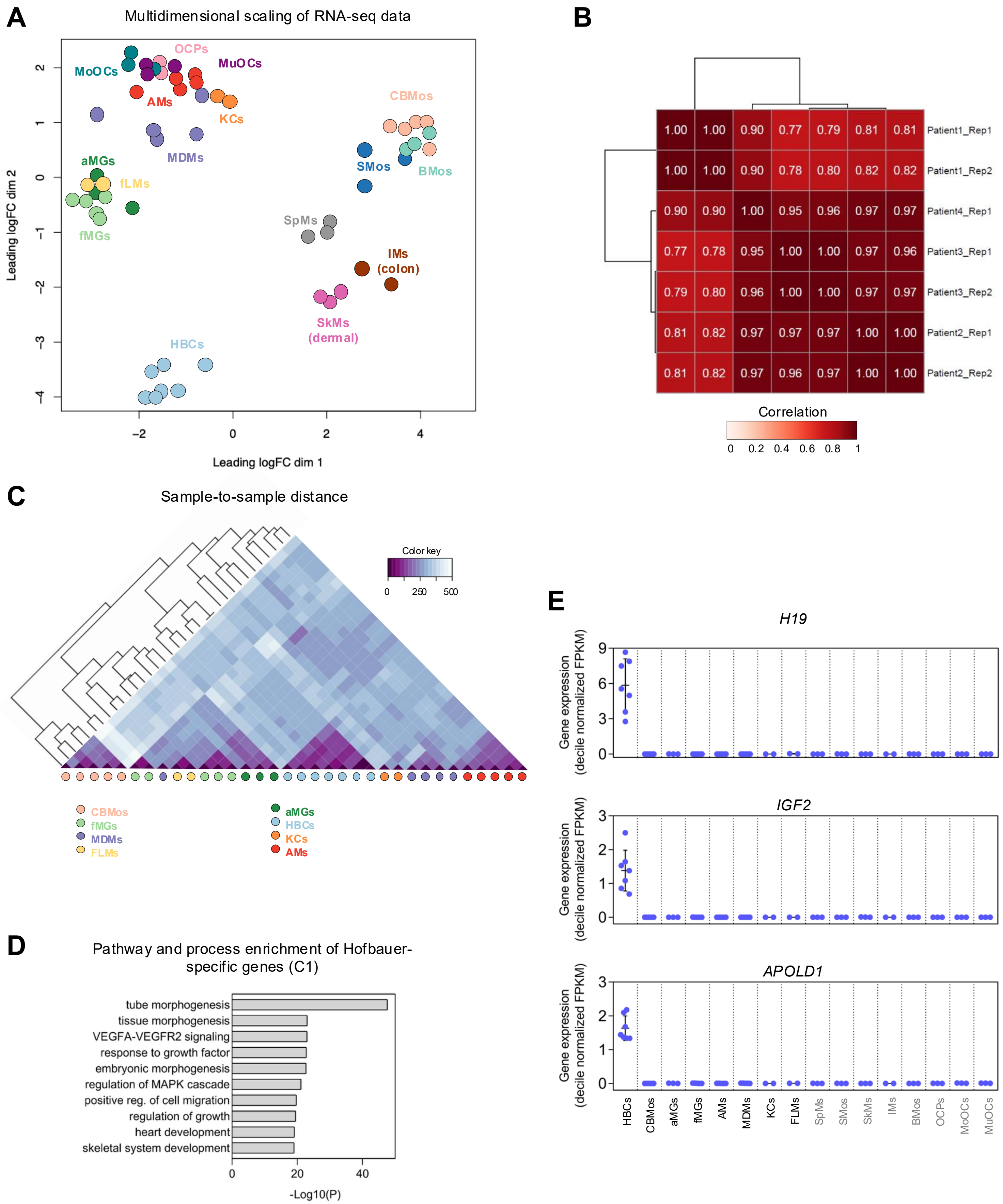

Supplementary Figure 4. Transcriptomic signature and functional enrichment of HBC–specific genes.
(A) The multidimensional scaling plot represents the Euclidean distances between the gene expression patterns of the 16 cell types indicated and also between their replicates. **(B)** The clustered heat map represents the Pearson correlation coefficients between technical replicates and across donors for RNA-seq samples. **(C)** The sample-to-sample distance heat map shows the similarity between the 8 cell types indicated and their replicates. **(D)** The bar chart depicts the biological pathway and process enrichments of HBC-specific genes (n=1413) from C1. **(E)** The scatter plots represent the relative normalized gene expression of the *H19*, *IGF2*, and *APOLD1* genes in the 16 cell types.

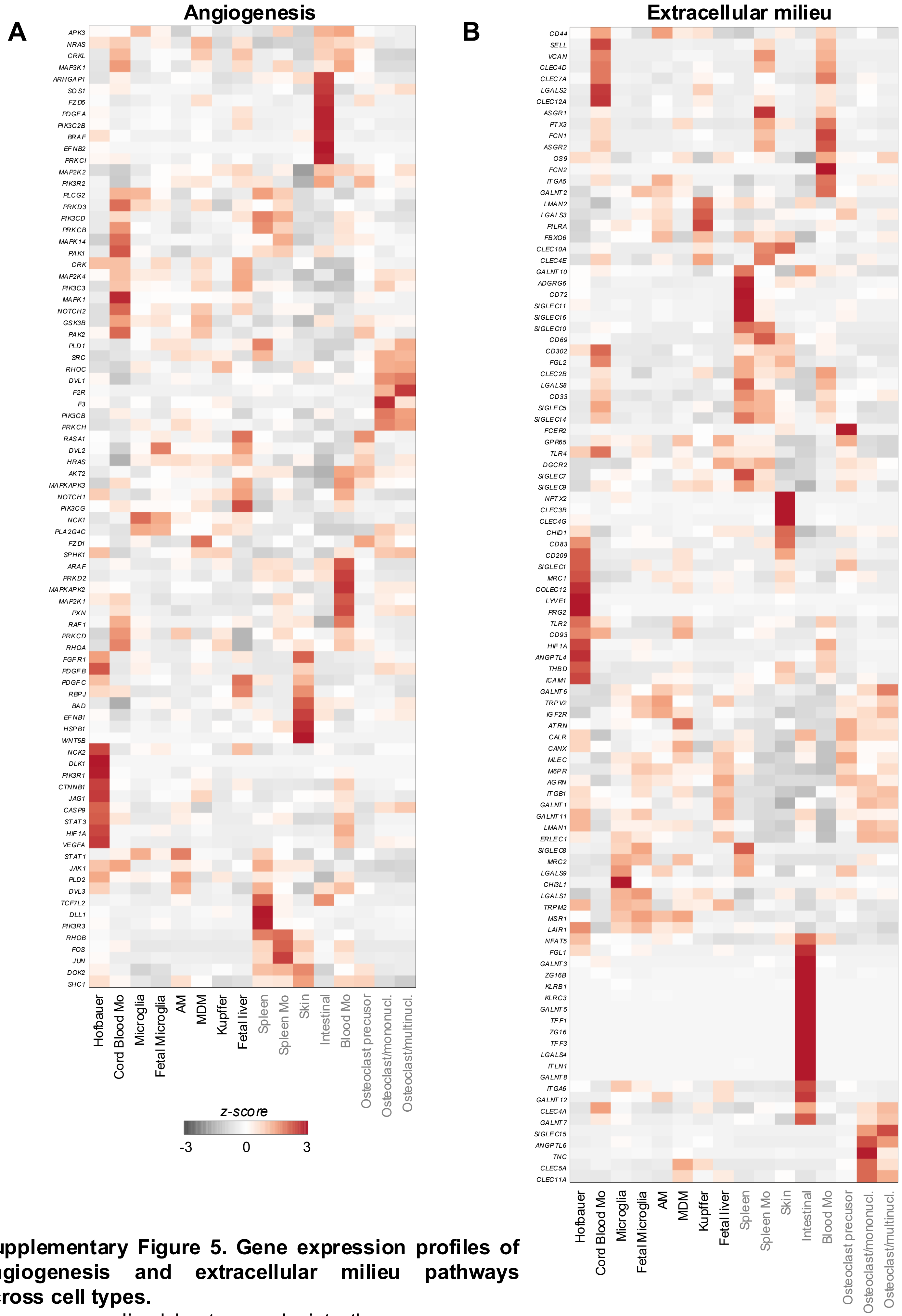

Supplementary Figure 5. Gene expression profiles of angiogenesis and extracellular milieu pathways across cell types.

The row-normalized heat map depicts the average gene expression of the **(A)** Angiogenesis-related and **(B)** Extracellular milieu genes in the 16 cell types indicated.

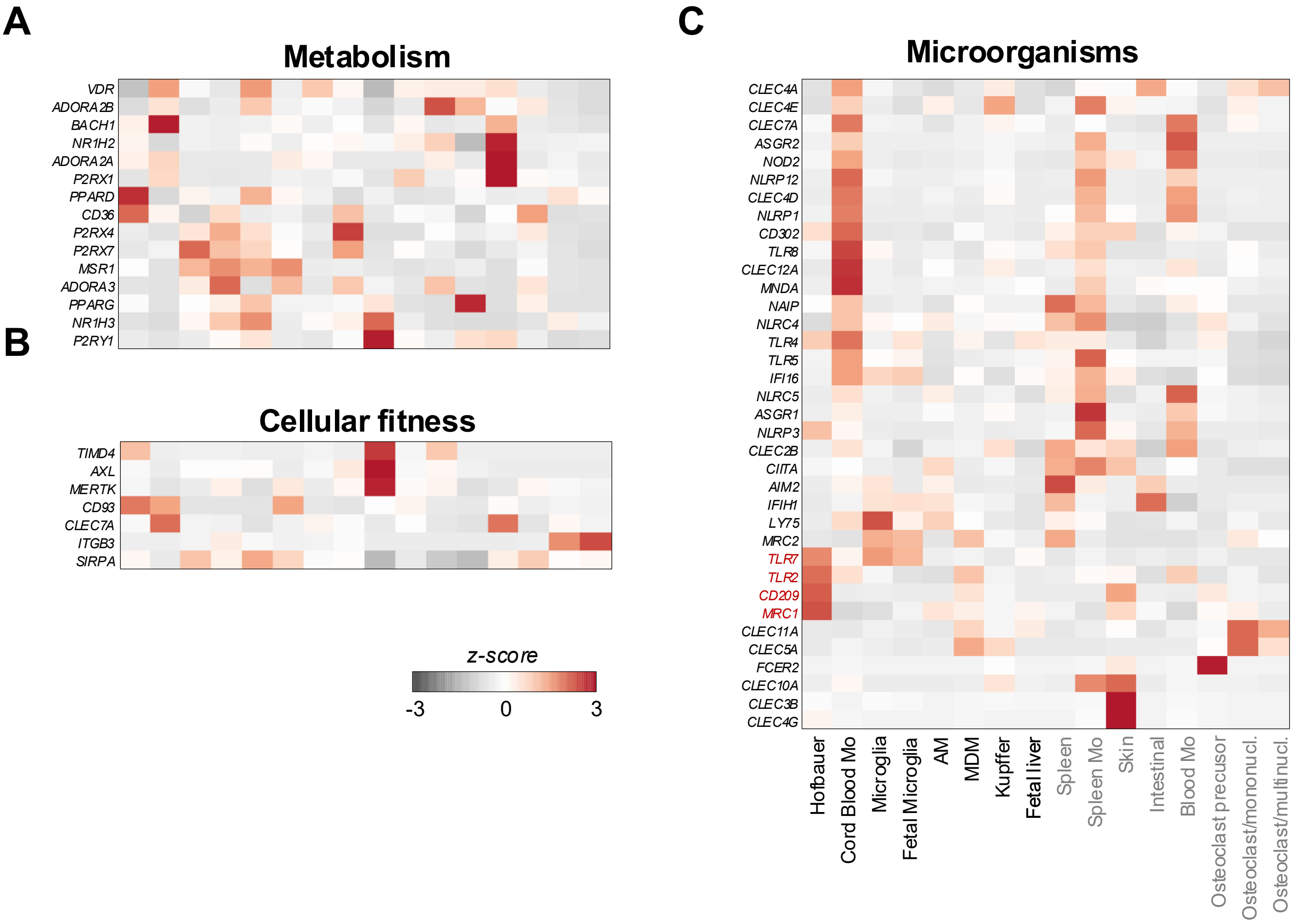

Supplementary Figure 6. Cell Type-Specific Expression of Genes Related to Metabolism, Cellular Fitness, Microbial Recognition, and Glucocorticoid Responsiveness.
The row-normalized heat maps depict the average gene expression of the **(A)** Microorganisms, **(B)** Metabolism-, or **(C)** Cellular fitness-related genes in the 16 cell types indicated

A

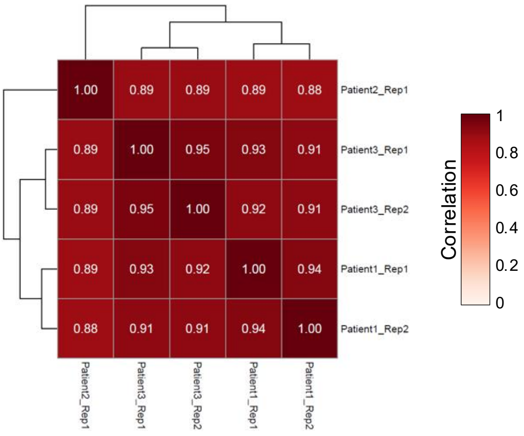

B

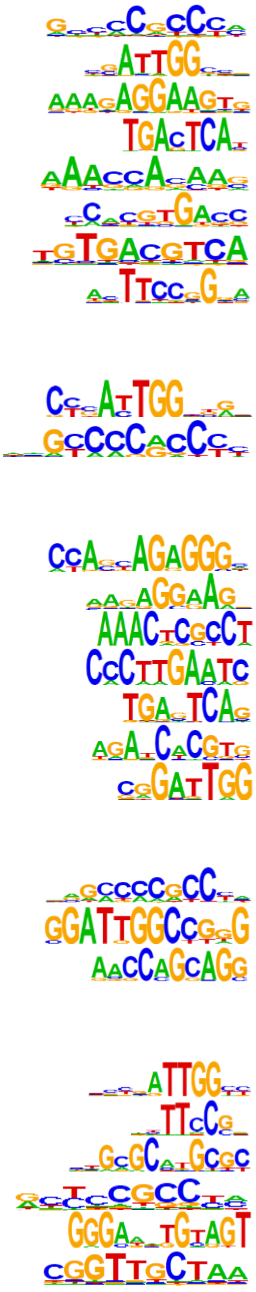

Promoters

P-value	Target %	Bg%	Motif
**MDM-specific (n=1110)**			
1e-50	33.30%	14.70%	**GC-box**
1e-29	13.42%	4.47%	**CCAAT-box**
1e-25	6.18%	1.20%	**PU.1**
1e-25	7.99%	2.02%	**TRE**
1e-16	5.23%	1.34%	**RUNX**
1e-12	4.28%	1.13%	**M-box**
1e-10	5.71%	2.19%	**CRE**
1e-10	11.04%	5.85%	**ETS**
**AM-specific (n=319)**			
1e-18	17.94%	4.30%	**CCAAT-box**
1e-14	25.91%	9.98%	**GC-box**
**Hofbauer- and MDM-specific (n=205)**			
1e-13	9.38%	0.84%	**CTCF**
1e-12	30.73%	11.19%	**PU.1**
1e-12	5.21%	0.15%	**Unknown**
1e-11	5.21%	0.19%	**NKX**
1e-10	18.75%	5.05%	**TRE**
1e-10	9.38%	1.26%	**E-box**
1e-10	20.83%	7.57%	**CCAAT-box**
**MDM- and AM-specific (n=217)**			
1e-13	26.94%	8.20%	**GC-box**
1e-29	4.15%	0.10%	**CCAAT-box**
1e-10	7.25%	0.81%	**Unknown**
**Hofbauer and AM-specific (n=1336)**			
1e-46	14.79%	4.45%	**CCAAT-box**
1e-39	31.57%	16.67%	**ETS-box**
1e-28	11.49%	4.08%	**NRF1**
1e-27	23.68%	12.58%	**GC-box**
1e-23	4.06%	0.67%	**GFY**
1e-145	9.12%	3.96%	**RFX**

C

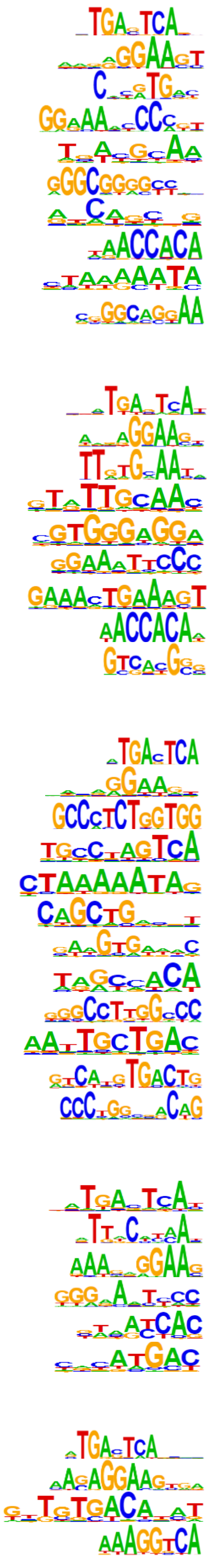

Enhancers (top 1,000)

P-value	Target %	Bg%	Motif
**MDM-specific**			
1e-139	27.09%	3.31%	**TRE**
1e-96	31.89%	7.61%	**PU.1**
1e-31	37.37%	19.98%	**M-box**
1e-24	8.91%	2.14%	**NFkB**
1e-22	26.17%	13.49%	**CRE**
1e-18	8.57%	2.58%	**GC-box**
1e-16	20.34%	10.61%	**AP4**
1e-14	8.11%	2.76%	**RUNX**
1e-13	2.63%	0.33%	**MEF2**
1e-12	7.66%	2.79%	**E2F (ETS?)**
**AM-specific**			
1e-76	27.34%	5.50%	**TRE**
1e-58	33.53%	10.59%	**PU.1**
1e-54	33.81%	11.28%	**CEBP**
1e-26	18.99%	6.62%	**CEBP:ATF**
1e-22	24.03%	10.74%	**EGR**
1e-18	11.51%	3.61%	**NFkB**
1e-16	6.19%	1.28%	**IRF**
1e-10	8.78%	3.62%	**RUNX**
1e-10	4.60%	1.51%	**PAX**
**Hofbauer- and MDM-specific**			
1e-144	39.06%	7.95%	**TRE**
1e-103	37.60%	10.18%	**PU.1**
1e-31	4.38%	0.29%	**CTCF**
1e-24	15.71%	5.93%	**ARE**
1e-24	13.47%	4.61%	**MEF2**
1e-19	32.10%	19.30%	**AP4**
1e-18	7.18%	1.92%	**PU.1:IRF**
1e-17	31.87%	19.70%	**RUNX**
1e-16	6.62%	1.81%	**DR0/DR1**
1e-15	5.61%	1.37%	**MARE**
1e-14	3.82%	0.70%	**MITF**
1e-13	7.86%	2.82%	**EBF?**
**MDM- and AM-specific**			
1e-122	26.31%	2.94%	**TRE**
1e-63	39.73%	14.47%	**CEBP**
1e-47	27.79%	9.20%	**PU.1**
1e-21	26.71%	13.39%	**NFkB**
1e-20	33.42%	19.01%	**RUNX**
1e-19	36.38%	21.55%	**M-box**
**Hofbauer and AM-specific**			
1e-44	14.22%	2.97%	**TRE**
1e-42	11.02%	1.82%	**PU.1**
1e-18	7.35%	1.84%	**MEIS**
1e-16	12.91%	5.34%	**NR4A**

Supplementary Figure 7. Transcription factor motif enrichment patterns in promoter and enhancer regions accross cell

(A) The clustered heat map represents the Pearson correlation coefficients between technical replicates and across donors for ATAC-seq samples. The *de novo* motif hits of **(B)** all the TSS-proximal (promoter) and **(C)** the top 1,000 TSS-distal (enhancer) regions of the C2-C6 clusters represented in Figure 4. The number of targeted promoters is highlighted in all cases.

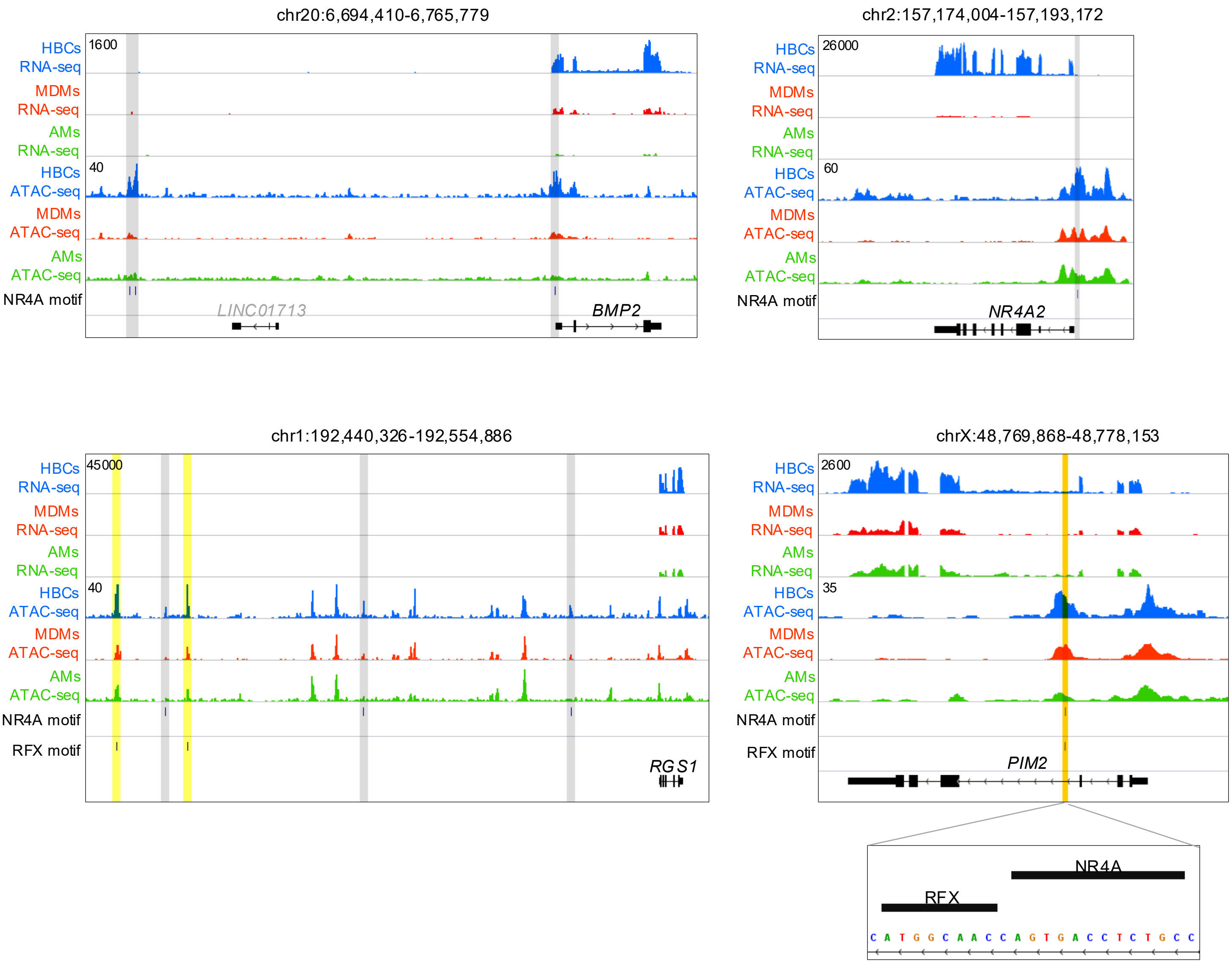

Supplementary Figure 8. NR4A and RFX motifs mark HBC-specific gene expression and chromatin accessibility

The IGV genome browser view of representative NR4A and RFX target genes, showing RNA-seq and ATAC-seq coverages from HBCs, AMs, and MDMs (replicates are overlaid). These genes display significantly higher expression in HBCs, where NR4A and RFX motifs are located within more accessible chromatin regions. NR4A motifs are marked in grey and RFX motifs in yellow. An example highlighted in orange shows an NR4A and RFX motif positioned directly next to each other. The interval scales are highlighted in all cases.

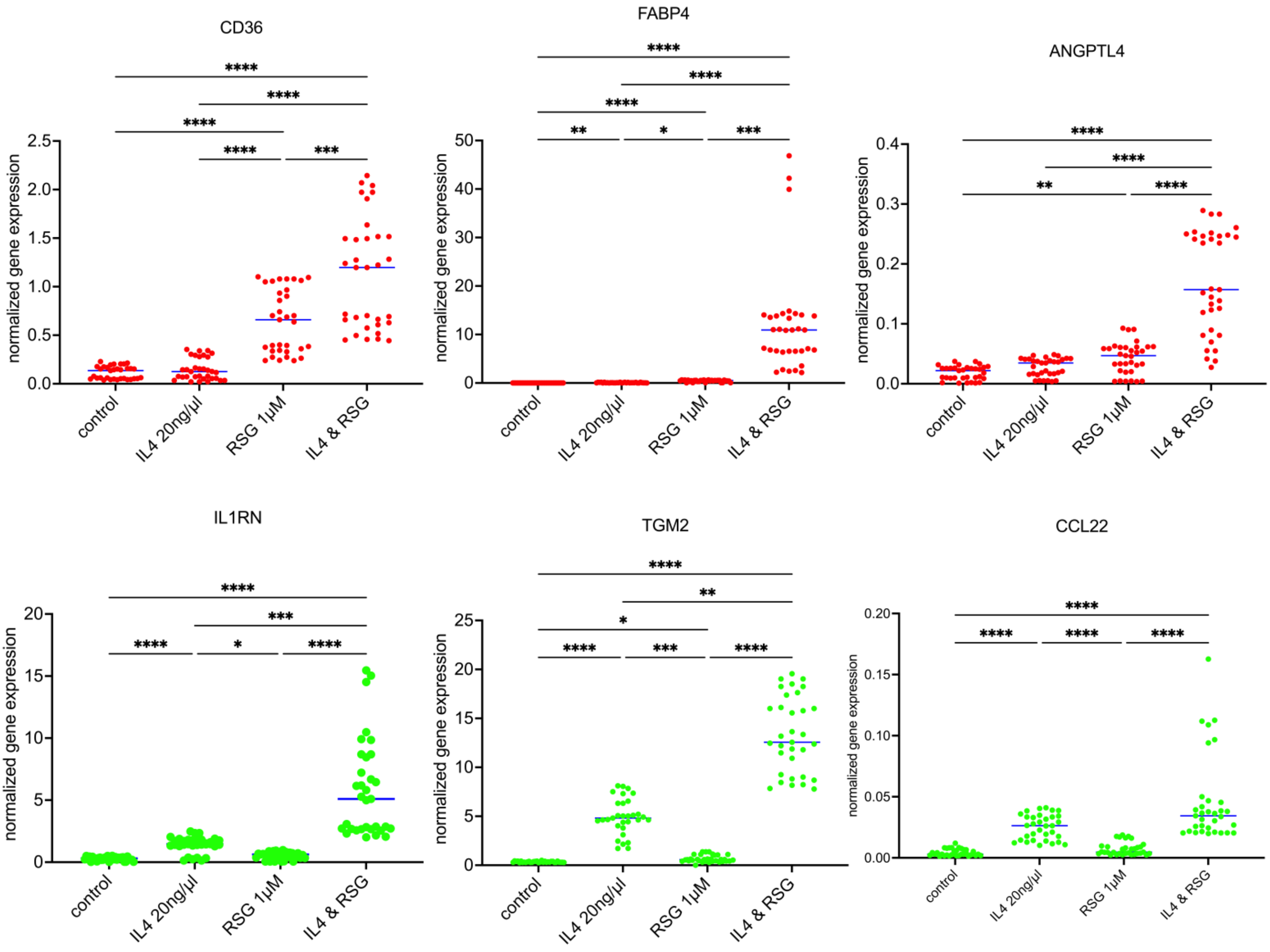

Supplementary Figure 9. *In vitro* stimulation assays with rosiglitazone and IL-4
Normalized mRNA expression levels after *in vitro* RSG and IL-4 induction, measured using PCR. Canonical PPARγ target genes are in red, and IL-4 induced genes are in green. Blue lines represent the medians of individual values. Pairwise comparisons were made using Kruskal-Wallis tests followed by Dunn's post hoc tests. Figure depicts replicates from the cells of 4 placentas. 1 dot represents 1 parallel measurement. Canonical PPARγ target genes are indicated in red, canonical IL-4 induced genes are indicated in green.
Statistical significance is indicated as follows: $p < 0.05$ (*), $p < 0.01$ (**), $p < 0.001$ (***), $p < 0.0001$ (****).

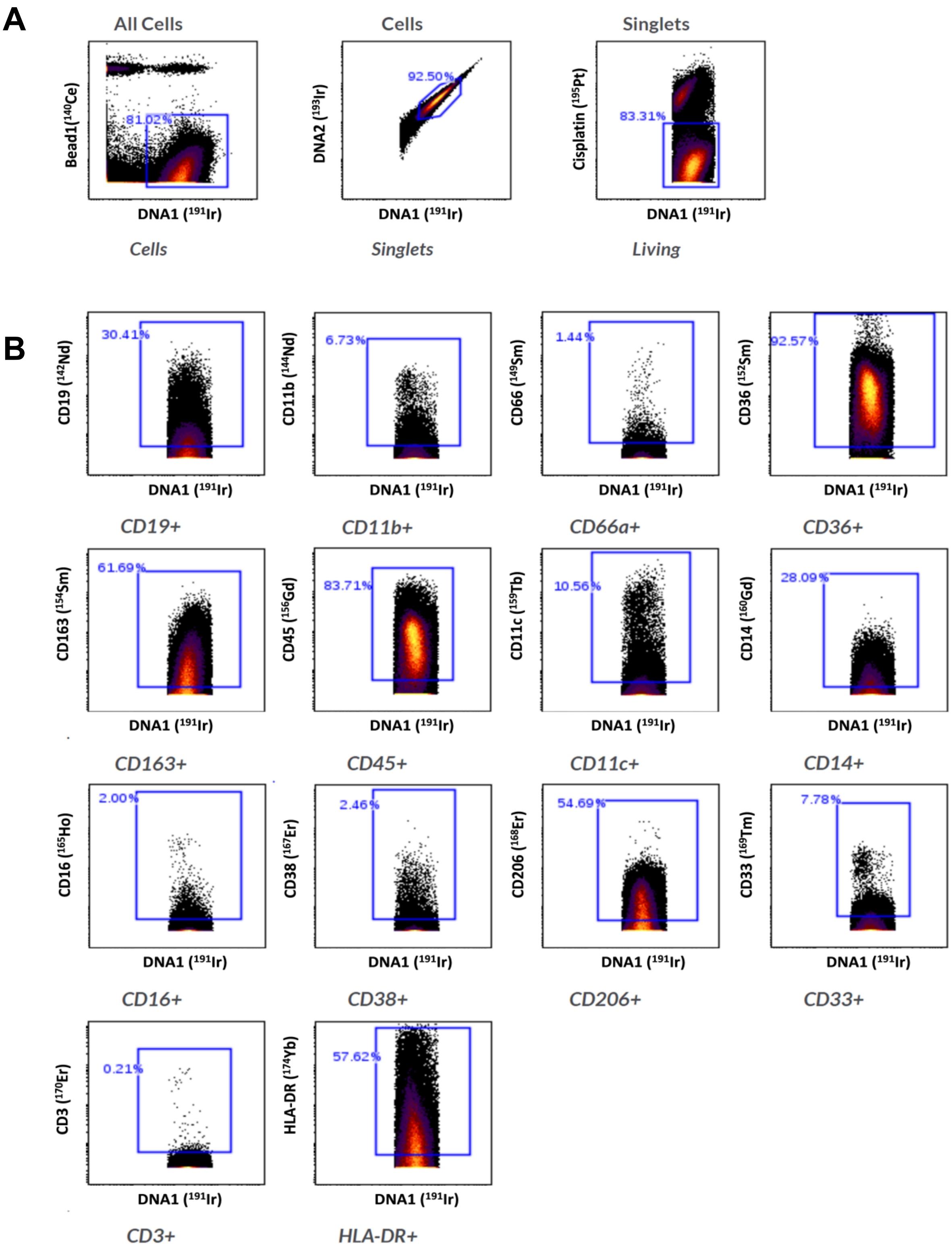

Supplementary Figure 10. Manual gating strategy of mass cytometry data.
(**A**) Cells, single cells (singlets), and living singlets were gated in Cytobank. (**B**) Gating strategy to define positivity for the investigated 14 markers.
